# Bacterial community assembly from cow teat skin to ripened cheeses is influenced by grazing systems

**DOI:** 10.1038/s41598-017-18447-y

**Published:** 2018-01-09

**Authors:** Marie Frétin, Bruno Martin, Etienne Rifa, Verdier-Metz Isabelle, Dominique Pomiès, Anne Ferlay, Marie-Christine Montel, Céline Delbès

**Affiliations:** 1 Université Clermont Auvergne, INRA, UMR545 Fromage, 20 côte de Reyne, F-15000 Aurillac, France; 2 Université Clermont Auvergne, INRA, VetAgro Sup, UMR1213 Herbivores, F-63122 Saint-Genès-Champanelle, France

## Abstract

The objectives of this study were to explore bacterial community assembly from cow teat skin to raw milk cheeses and to evaluate the role of farming systems on this assembly using 16S rRNA gene high-throughput sequencing. The two grazing systems studied (extensive vs. semi-extensive) had a greater effect on the microbiota of cow teat skin than on that of raw milks and cheeses. On teat skin, the relative abundance of several taxa at different taxonomic levels (*Coriobacteriia*, *Bifidobacteriales*, *Corynebacteriales*, *Lachnospiraceae*, *Atopobium*, and *Clostridium)* varied depending on the grazing system and the period (early or late summer). In cheese, the abundance of sub-dominant lactic acid bacteria (LAB) varied depending on the grazing system. Overall, 85% of OTUs detected in raw milks and 27% of OTUs detected in ripened cheeses were also found on cow teat skin. Several shared OTUs were assigned to taxa known to be involved in the development of cheese sensory characteristics, such as *Micrococcales*, *Staphylococcaceae*, and LAB. Our results highlight the key role of cow teat skin as a reservoir of microbial diversity for raw milk, and for the first time, that cow teat skin serves as a potential source of microorganisms found in raw-milk cheeses.

## Introduction

The microbial community of cow teat skin depends on the dairy farm environment^1^. Microorganisms can colonize the teat skin through contact with the bedding material, a factor that depends on animal feeding and housing conditions^1,2^. The microbiota of forages fed to dairy cows is obviously related to the preservation method used (grazed grass, hay or silage). It may vary also according to the characteristics of the grasslands that create more or less favourable conditions (for example, relative humidity, temperature, and ultraviolet radiation) for the colonisation of the phyllosphere by bacteria, yeasts, and filamentous fungi^3^. In addition, the composition of animal feed is known to affect the structure of the microbial community in the rumen as well as in the faeces^4,5^. Aerosolized dust within the animals’ stables and hygiene practices during milking (e.g. washing of milking equipment, pre- and post-milking teat care) are also potential sources of bacteria and fungi present on the teats^6,7^. The dairy farm environment and farming practices and their influence on the milk microbiota have therefore become a subject of interest for researchers studying cheese microbial ecology. In addition, it is not known whether differences in cheese sensory characteristics attributed to grassland and animal feeding characteristics^8,9^ may be associated with differences in the composition of the teat skin and milk microbiota^10^.

The teat skin of cows is considered as a major reservoir of microbial diversity of raw milk; many bacterial genera found in raw milk are also detected on teat skin^1,6,11^. This ecosystem is inhabited by many microorganisms which may also grow in cheeses during ripening, such as lactic acid bacteria, staphylococci, and corynebacteria.

The composition of the milk microbiota depends on the microbial composition of different reservoirs directly in contact with the milk, such as the teat skin of cows and dairy equipment including milking machines, milk lines, and bulk tanks. In addition to starters and ripening cultures, the cheese-making and ripening equipment and environments further influence the microbial composition of raw-milk cheeses^12,13^. The microbiota of raw milk has a pivotal role in the development of cheese flavour, as well as implications for shelf life and safety^14,15^.

Recent advances in high-throughput sequencing (HTS) provide a powerful means to analyse dominant and subdominant populations and their dynamics in complex ecosystems^16,17^. The rDNA-based metabarcoding approach offers the opportunity to monitor Operational Taxonomic Units (OTUs) across successive habitats (teat skin, milk, cheese, etc.). Using HTS, Doyle *et al*.^5^ identified teat surfaces and faeces as the most prevalent sources of microorganisms in raw milk, but to our knowledge, no studies to date have shown a direct link between the microbiota of teat skin and that of raw-milk cheeses.

The aim of this work was to explore bacterial community assembly from cow teat skin to ripened raw-milk cheese and to test the hypothesis that the choice of two contrasted grazing systems, (extensive (EXT) and semi-extensive (SEMI)), could serve as a lever to shift the microbial balance of raw milk and cheese *via* the microbiota of teat skin. We investigated the bacterial populations of teat skin, raw milk, and cheese rind and core using high-throughput 16S rRNA gene sequencing.

## Results

### Bacterial composition of cow teat skin swabs and dairy product samples

**Figure 1:**
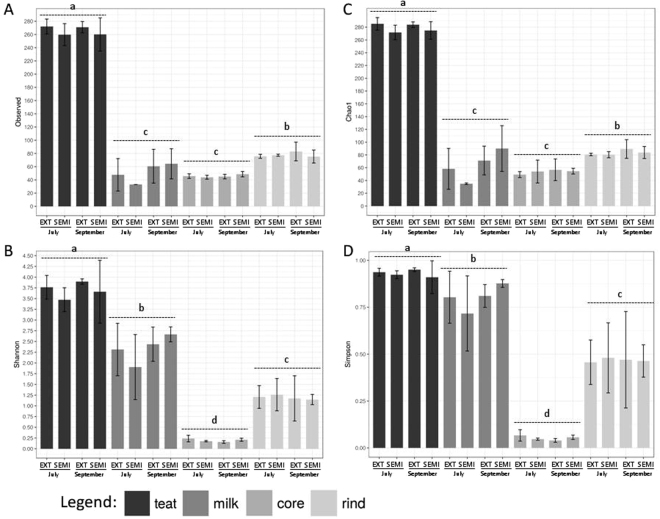
Alpha-diversity inferred from 16S rRNA sequence data: number of observed OTUs (**A**), species richness (Chao1) (**B**), diversity indexes Shannon (**C**) and Simpson (**D**) in the four habitats (teat skin, raw milk, cheese core, and cheese rind) according to period (July and September) and grazing system (EXT and SEMI). Values are means of triplicate (*n* = *3*) and bars in the columns represent the mean standard error. Habitats with different letters were significantly different (LSD test, *P* < 0.001).

A total of 1,636,464 reads from 48 samples were distributed to 365 OTUs after filtration steps. The species richness (Chao1) and diversity (Simpson and Shannon indices) were calculated for each grazing system and each period of cheese-making in each habitat (Fig. 1). The values were significantly different (P < 0.001) depending on the habitat. The diversity indices revealed decreased diversity from teat skin to ripened cheeses as well as a higher diversity on cheese rind than in cheese core. The habitat had a greater effect than the grazing system and the period of cheese-making on the bacterial composition. The species richness and diversity did not differ significantly according to the grazing system or the period of cheese-making in any habitat.

**Figure 2:**
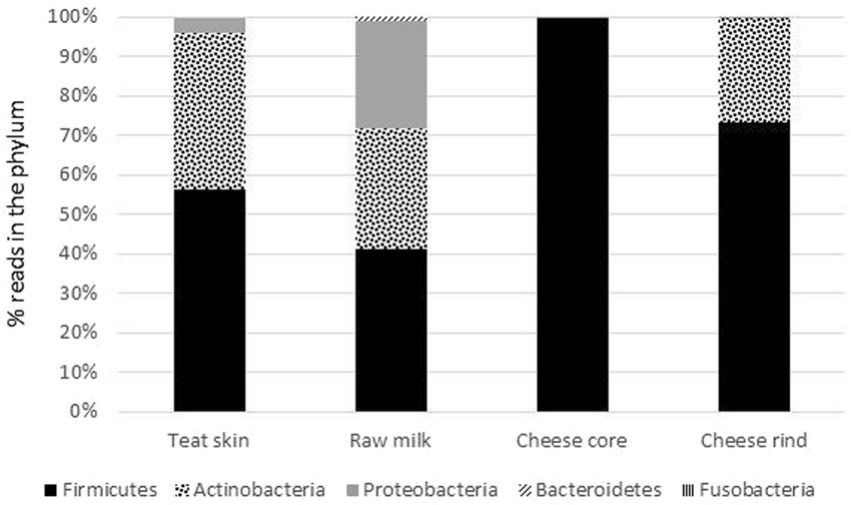
The bacterial composition at phylum level in four habitats.

**Figure 3:**
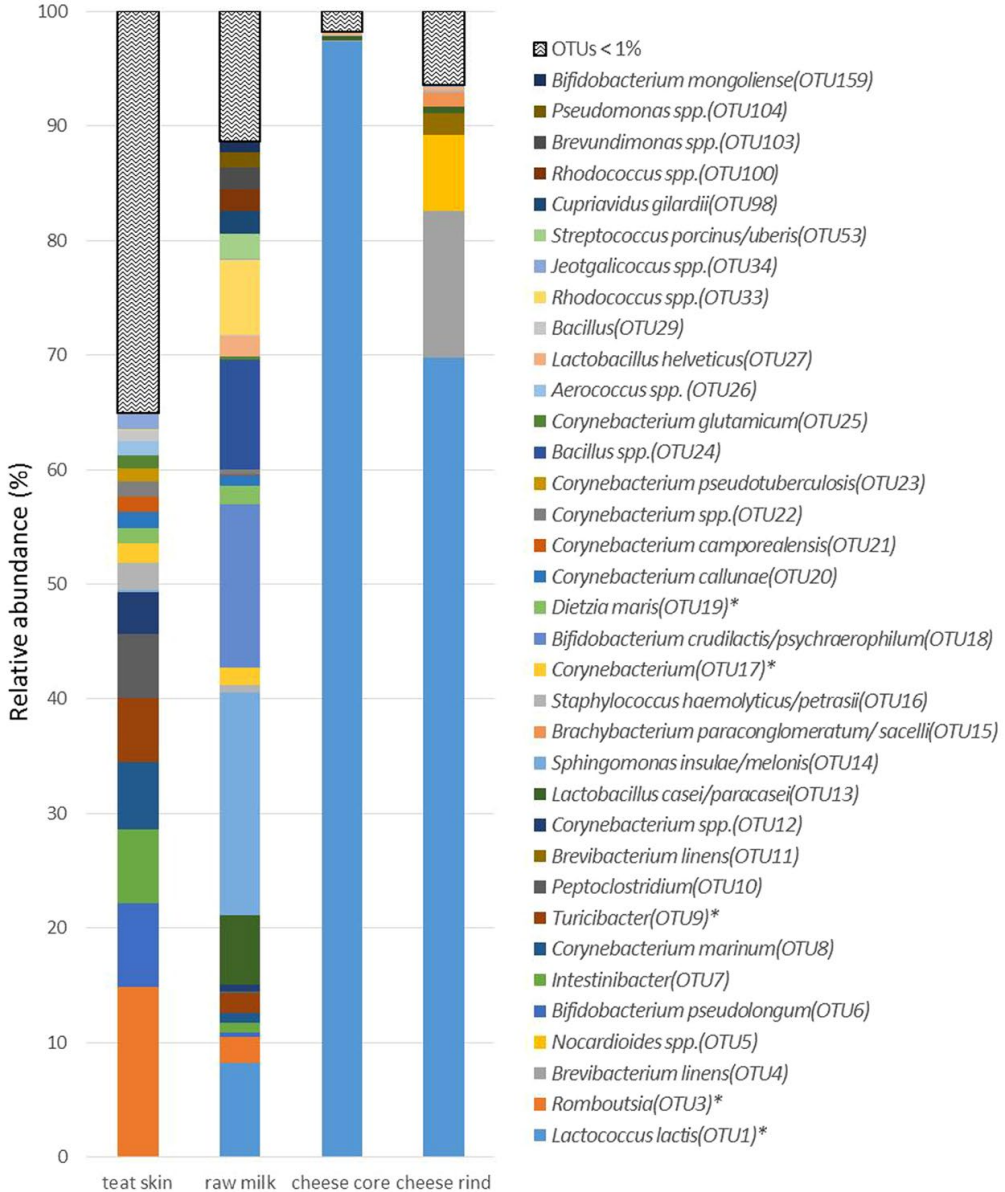
Relative abundance of 34 OTUs present at more than 1% in at least one of the habitats. The * symbol indicates the shared OTUs between two or more habitats.

The predominant phylum in all habitats was *Firmicutes*, particularly in the cheese core (>99% of the total bacterial reads; Fig. 2), followed by *Actinobacteria*, for which relative abundance decreased slightly from teat skin to cheese rind. The phylum *Proteobacteria* was very abundant in milk compared to other habitats. The highest number of OTUs was found on teat skin (in total, 300 OTUs including 98 bacterial genera; Supplementary Table S1). Among the dominant OTUs (>1%) on teat skin, the 4 OTUs assigned to *Romboustia* (OTU3), *Turicibacter* (OTU9), *Corynebacterium* (OTU17) and *Dietzia maris* (OTU19) also had a high abundance in raw milk (Fig. 3). The lactic acid bacteria *Lactococcus* and *Lactobacillus* were predominant both in milk and in core and rind of cheese, while the microbiota of teat skin was dominated by the genus *Corynebacterium*. The dominant OTUs on cheese rind were assigned to ripening bacteria (*Brevibacterium linens*, *Nocardiopsis* spp. and *Brachybacterium paraconglomeratum/sacelli*) and to *Lc*. *lactis*, together representing 92.2% of total abundance (Fig. 3).

The proportion of phyla among isolates obtained from raw milk samples and identified by sequencing of the 16S rRNA gene was similar to that found using the metabarcoding approach (Supplementary Table S2). However, an additional phylum corresponding to *Deinococcus-Thermus* was detected in EXT milks. In total, 34 bacterial genera were identified, of which 12 were not detected by metabarcoding (genera underlined in Supplementary Table S2), especially in the phylum *Actinobacteria*.

### Effect of grazing system on OTUs abundance profiles in the different habitats from teat skin to ripened cheeses

**Figure 4:**
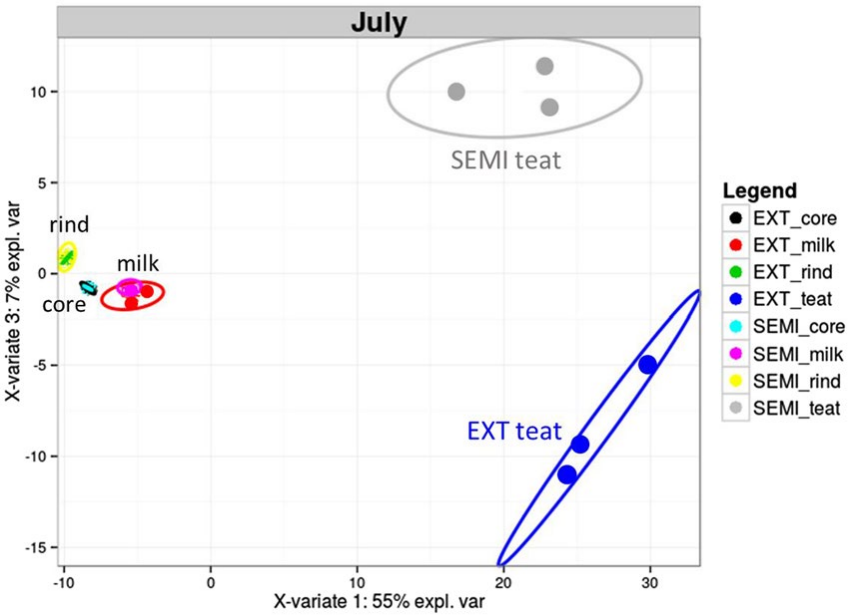
Partial least squares discriminant analysis (PLS-DA) performed on the relative abundance of 365 OTUs found in 24 samples of teat skin and dairy products (milk, cheese core, and cheese rind) from EXT and SEMI grazing systems in July. Plot of sample distribution is projected on principal components 1 and 3 (PC1 and PC3).

A PLS-DA was performed to identify the OTUs that contributed the most to the variance between samples originating from each grazing system for every habitat considered. The bacterial composition of teat skin was clearly different from that of dairy products at both periods. In July, the 80 OTUs that contributed the most to the PC3 discriminating the EXT from the SEMI systems were identified in teat skin samples (Fig. 4 and Supplementary Fig. S1). In September, the OTUs with a differential abundance between EXT and SEMI systems were also detected on cow teat skin, although they were not among those who contributed the most to the PC3 (Supplementary Fig. S2).

**Figure 5:**
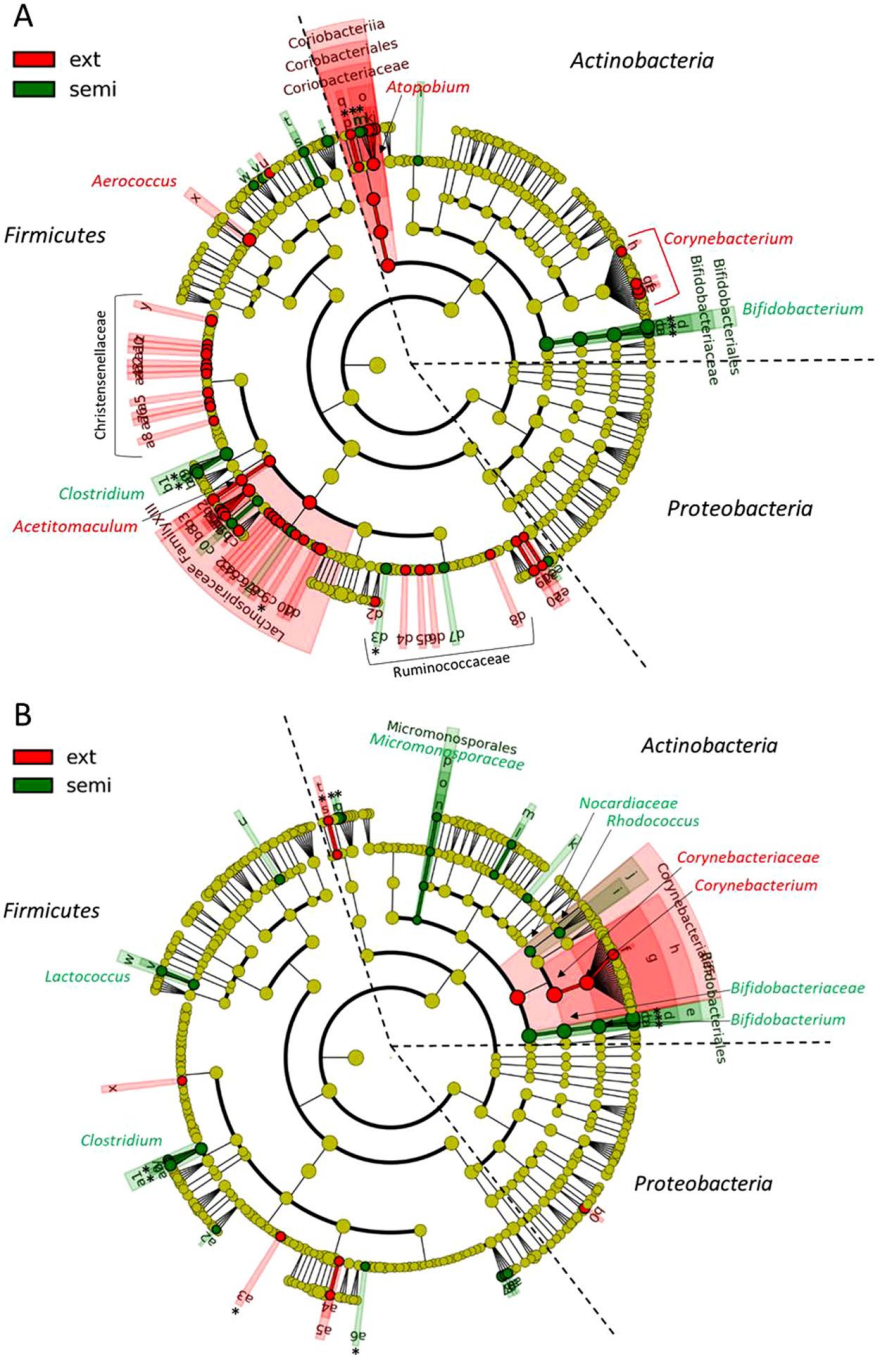
LEfSe cladogram demonstrating taxonomic differences in cow teat skin microbiota between the two grazing systems (EXT and SEMI) **(A)** in July, and **(B)** in September. Taxa and nodes highlighted in red and green were significantly more abundant in EXT and SEMI systems, respectively. The diameter of each circle is proportional to the abundance of the taxon. Only differentially abundant taxa at the genus or higher taxonomic ranks were indicated. For details on differentially abundant OTUs, see Tables S2 and S3. Nodes remaining light green indicate taxa that were not significantly differentially represented. The symbol * indicates identical taxa with a differential abundance at both periods.

A linear discriminant analysis (LDA) on teat skin samples pointed out multiple significant differences in the relative abundance of bacterial taxa depending on grazing system at the two periods. Significant differences observed at the genus level or at higher taxonomic ranks can be seen in Fig. 5. Complete results of discriminant analysis down to the OTU, corresponding to the LDA score and *p-value*, are included in Supplementary Tables S3 and S4. Differences concerned almost exclusively taxa assigned to the *Actinobacteria* and *Firmicutes* phyla, with the exception of one sub-dominant OTU assigned to *Proteobacteria*. At both periods studied, compared to that of EXT cows, the microbiota on teat skin of cows reared in the SEMI system was characterized by a 14 to 22-fold higher relative abundance, in September and July respectively, of the order *Bifidobacteriales* (LDA score >4.9, abundance up to 21% in September) including *B*. *pseudolongum* (OTU6), *B*. *choerinum* (OTU36), and *B*. *merycicum* (OTU52). Among sub-dominant taxa, a 2- to 4-fold higher abundance of the genus *Clostridium* (abundance up to 2.3% in July) including *Clostridium* spp. (OTU35) was noticed on teat skin of SEMI cows. Conversely, the microbiota on teat skin of EXT cows was characterized by a 2- to 4-fold higher relative abundance of the genus *Corynebacterium* (LDA score >4.9, abundance up to 31% in September) including *Corynebacterium* spp. (OTU12) (up to 3.9% in July). In July, there were a total of 71 bacterial taxa with a significantly different relative abundance (*P* < 0.05) on teat skin of EXT and SEMI cows (Fig. 5A and Supplementary Table S3). The microbiota on teat skin of EXT cows was characterized by a 3-fold higher relative abundance of the family *Lachnospiraceae* (LDA score >4.2, abundance up to 4.6%) including the genus *Acetitomaculum* and of the class *Coriobacteriia* (abundance up to 1.7%) including the genus *Atopobium*. Likewise, the genus *Aerococcus* had a 6-higher abundance on teat skin of EXT cows (abundance up to 2.4%) than that of SEMI cows. The number of significantly different taxa in September (*P* < 0.05) was half that of July and they were not necessary the same (Fig. 5B and Supplementary Table S4). The microbiota on teat skin of EXT cows was characterized by a 2-higher relative abundance of the order *Corynebacteriales* (LDA score >4.9, abundance up to 34%) and by a 23-fold lower abundance of the sub-dominant taxa *Atopobium* (OTU75) (abundance up to 1.1%) compared to that of SEMI cows.

In raw milks, cheese cores, and cheese rinds, OTUs with differential abundance between EXT and SEMI systems were less numerous than on teat skin and their relative abundance was generally below 1% both in July and in September (Supplementary Tables S5, S6 and S7). Yet differences in abundance of higher taxonomic ranks were highlighted. In July, compared to that of SEMI cows, the raw milk microbiota of EXT cows was characterized by a 25-fold higher relative abundance of the class *Gammaproteobacteria* (LDA score >4.3, abundance up to 3.4%) including the order *Pseudomonadales* and by a 6-fold higher abundance of the family *Lachnospiraceae* as a sub-dominant taxon (Supplementary Table S5A). In terms of the cheeses, both the core and the rind microbiota of EXT cheeses were characterized by a 2- to 4-fold higher relative abundance of the family *Lactobacillaceae* (LDA score >4, abundance up to 1.5%) including *Lactobacillus helveticus* (OTU27 and 229) (Supplementary Tables S6A and S7A). In September, the microbiota in raw milk of SEMI cows was characterized by a 2-higher relative abundance of the phylum *Firmicutes* (LDA score >5, abundance up 48%; Supplementary Table S5B). In cheeses made from this milk, the sub-dominant OTUs (abundance up to 0.36%) assigned to *Lactococcus lactis* (OTU46), *Lactococcus chungangensis/raffinolactis* (OTU48) and *Lactococcus* (OTU89) were 4- to 5-fold more abundant than in EXT cheeses, both in core and rind (Supplementary Tables S6B and S7B). Conversely, among sub-dominant taxa, a 4-fold higher abundance of *Rhodococcus* spp. (OTU100) (abundance up to 3.2%) was observed in EXT milk (Supplementary Table S5B).

### Network analysis of the bacterial OTUs across the successive habitats from teat skin to ripened cheeses

**Figure 6:**
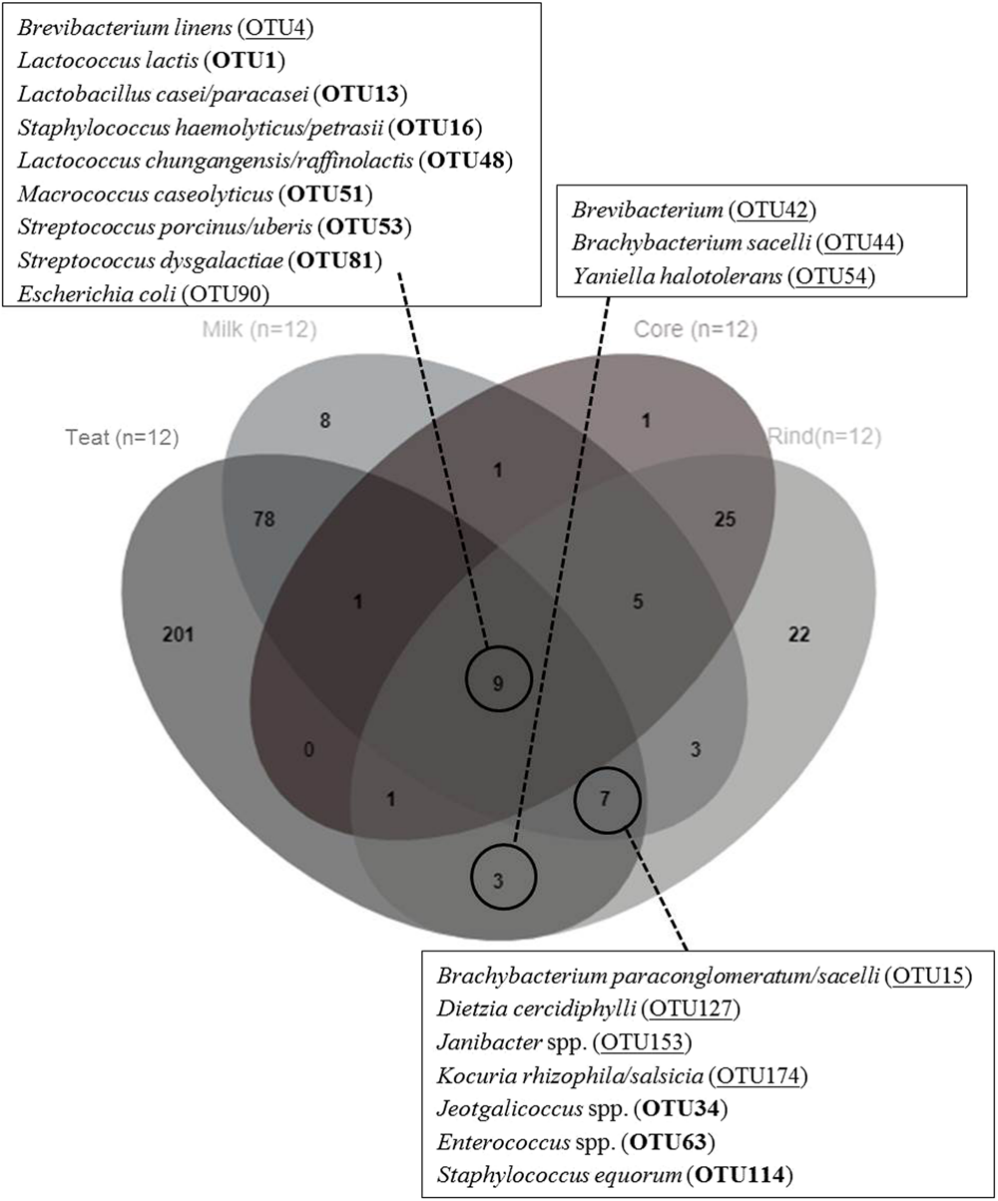
Venn diagram: occurrence of the 365 OTUs across the habitats from teat skin to ripened cheeses. The OTUs detected in at least two samples of the same habitat were conserved. The underlined OTUs were assigned to the phylum *Actinobacteria* and those in bold to the phylum *Firmicutes*.

The OTU network clearly showed differences between the teat skin, milk and cheese core/rind samples whatever the grazing system and period. The Venn diagram indicated specific and shared OTUs from teat skin to ripened cheeses (Figs 6 and S3). The relative abundance of the 133 OTUs found in at least two habitats was detailed in Supplementary Table S8. Among the 300 OTUs detected on teat skin, one third (99 OTUs) were also found in dairy products, mainly in raw milk (95 of the 112 OTUs detected in milks). Among the total 78 OTUs detected both in core and rind of cheeses, 21 were also detected on teat skin, with a majority of OTUs assigned to *Firmicutes* (11 OTUs including *Lactobacillales* and *Staphylococcaceae*) followed by *Actinobacteria* (8 OTUs, mainly found on cheese rind) (Fig. 6). Nine of the OTUs detected in cheese were also identified in milk but not on teat skin (Supplementary Table S8). Among the 43 OTUs detected in cheese cores, the majority (40 OTUs) were also detected on cheese rind, of which 26 OTUs were neither found on teat skin and nor in milk. The latter comprised 19 OTUs assigned to lactic acid bacteria (*Lactococcus* and *Lactobacillus*). There were 22 OTUs exclusively detected in cheese rinds, the majority of which were assigned to ripening bacteria (*Brevibacterium*, *Brachybacterium* and *Nocardiopsis*).

Nine OTUs were found in all habitats, including cheese rind and core. The relative abundance of *Lactococcus lactis* (OTU1), *Lactobacillus casei/paracasei* (OTU13) and *Lactococcus chungangensis/raffinolactis* (OTU48) increased from teat skin to dairy products (Supplementary Table S8). *Brevibacterium linens* (OTU4) had a higher relative abundance in cheese rind than in cheese core, milk, and teat skin. The relative abundance of *Staphylococcus haemolyticus/petrasii* (OTU16), *Macrococcus caseolyticus* (OTU51), *Streptococcus porcinus/uberis* (OTU53) and *Streptococcus dysgalactiae* (OTU81) was higher on teat skin and milk than in cheese. *Escherichia coli* (OTU90) had a very low abundance in the four habitats. In addition, 5 OTUs assigned to *Actinobacteria* (*Brevibacterium* (OTU42), *Brachybacterium paraconglomeratum/sacelli* (OTU15 and OTU44), *Yaniella halotolerans* (OTU54), *Dietzia cercidiphylli* (OTU127)) and detected on teat skin at very low abundance (<0.07%) showed an increased relative abundance on cheese rind (0.09% to 1.22%).

## Discussion

### Effect of grazing system on the bacterial balance on teat skin, in milk and in cheese

This experiment was designed to focus on the effect of grazing systems, since the dairy equipment (milking machine, milk line and tank) and the equipment and parameters of cheese-making were strictly identical. We made the assumption that the long-term system experiment (which began one year before the present work) allowed adaptation of the microbial community structure to each system.

The main differences found in the abundance of microbial taxa between the grazing systems concerned the teat skin microbiota. Faeces are recognized as a potential source of microorganisms on cow teat skin^11^. The faecal microbiota in cattle is affected by diet, particularly differences between forage- and concentrate-based diets^18^. These authors showed that the abundance of the genus *Clostridium* and the *Lachnospiraceae* and *Ruminococcaceae* families differed in faeces according to diet. In this study, we noticed a higher abundance of the genus *Clostridium* on teat skin of SEMI cows, which received concentrates. This daily distribution of concentrate to the SEMI cows in a collective feed trough could cause acidosis in the dominant cows, which may have ingested large amounts of concentrates. Indeed, the milk fat-to-protein-ratio was below the threshold of 1 for five SEMI cows in July and two SEMI cows in September (data not shown), suggesting that these SEMI cows were in subacute ruminal acidosis^19^ contrary to EXT cows. The drop of ruminal pH disrupts the microbiota of the rumen^5^ as well as the faecal microbiota^20^. The relative abundance of OTUs assigned to *B*. *choerinum*, *B*. *merycicum*, *B*. *pseudolongum*, *Atopobium* (OTU75 and 212), and *Ruminococcaceae* (OTU197) was higher on teat skin of SEMI than EXT cows. Although *Bifidobacterium* is typically associated with the gastrointestinal tracts of mammals, its abundance was much higher on teat skin of SEMI cows (abundance up to 21% in September). Similarly, Mao *et al*.^21^ reported a higher abundance of the *Bifidobacterium*, *Atopobium* and *Ruminococcus* genera in the rumen microbiota during acidosis. Despite their low abundance on teat skin, we found that the abundance of *Solibacillus* (OTU205) was lower, while that of *Turicibacter* (OTU711) was higher on teat skin of SEMI cows compared to EXT cows, in July. In another study, Mao *et al*.^20^ observed a similar result on the abundance of *Solibacillus silvestis* and *Turicibacter* in the faecal microbiota of cows in acidosis. The soil surface and phyllosphere microbiota of the grazed paddocks, especially in the grassland areas used by cows for bedding, could also be a major factor explaining the observed differences in bacterial balance. We can make the assumption that the higher botanical diversity of EXT grasslands (on average 86 plant species in EXT system and 36 in SEMI system)^22^ and the fertilization of SEMI grasslands can modify the abundance, diversity and activity of soil microbial communities as previously demonstrated^23,24^. Nevertheless, the relationships between phyllosphere and teat skin microbiota are not yet understood. The effect of grazing system was lower on teat skin microbiota in September than in July, and most OTUs with a differential abundance between EXT and SEMI cows were not the same in July and in September. The observed differences between July and September might be explained by the cow lactation stage and/or by environmental factors (e.g., climate, maturation stage of grass, botanical composition). Several authors reported an increase in the proportion of *Actinobacteria* in milk of cows and goats at late lactation possibly due to environmental conditions^25,26^.

The effect of grazing system was not as apparent in the raw milk microbiota. The taxa with a differential relative abundance in the different habitats were rarely the same apart from the family *Lachnospiraceae*, which had a higher overall abundance for EXT system than for SEMI system on both teat skin and in milk. We can hypothesize that the milking of all cows (EXT and SEMI systems) with the same milking machine on the same day had a “funnel effect” possibly related to the transfer to milk of micro-organisms originating from the biofilm adhering to the inner surfaces of milking pipes^7,11^. The addition of the same starter and ripening culture combined with the cheese-making and ripening in the same dairy facility could also explain the reduction of the bacterial differences between EXT and SEMI cheeses.

However, several findings of our study regarding LAB may be of particular importance for the dairy industry. We observed that the relative abundance of the family *Lactobacillaceae* including *Lb*. *helveticus* was higher in July both in core and rind of EXT cheeses than in those of SEMI cheeses, which was not the case in September. However, we are not able to explain this difference by environmental factors linked to the period. Lactobacilli are sought for their proteolytic activity and ability to produce aroma compounds^27^. In cheeses made in September, we observed that the relative abundance of *Lc*. *lactis*, *Lc*. *chungangensis/raffinolactis* and *Lactococcus* was slightly higher both in core and rind of SEMI cheeses than in those of EXT cheeses. We could hypothesize that the growth of starter culture (*Lactococcus lactis* ssp. *lactis*, *Lactococcus lactis* ssp. *cremoris*, *Lactococcus lactis* ssp. *lactis* biovar *diacetylactis*) may be modified as well. Nevertheless, the metabarcoding approach could not discriminate between bacteria at the strain level or specifically monitor starter strains, nor did it show whether these taxa were metabolically active. The primary role of lactococci during cheese production is acidification; however, they also contribute to proteolysis, to the conversion of amino acids into flavour compounds, and to fat metabolism^28,29^. Frétin *et al*.^22^ reported that the biochemical composition of milk, in particular that of fatty acids, was different in milk and cheeses originating from the EXT or the SEMI system. Free fatty acids can have adverse or favourable effects on bacterial growth depending on their nature and concentration^30,31^. Further work is needed to determine whether interactions between the biochemical and the microbial components of milk may explain differential abundance of non-starter and starter LAB in cheese depending on the grazing system.

### Bacterial community assembly from teat skin to ripened cheeses

In the present study, a metabarcoding approach was used, for the first time, to investigate the bacterial dynamics from cow teat skin to ripened cheeses. This HTS approach revealed that the teat skin of cows harbours a high diversity of bacteria. Compared to previous studies, the number and proportion of phyla detected here were different, even though the phylum *Firmicutes* always showed the highest abundance. Verdier-Metz *et al*.^1^ detected 46 bacterial genera/species by culture-dependent method and 78 bacterial genera/species by 16S rRNA gene clone libraries. Thanks to the high sequencing depth used in the present study, we identified 98 bacterial genera on cow teat skin encompassing 300 OTUs.

Nine OTUs were detected all along the path from teat skin to ripened cheeses. These OTUs are assigned to seven genera (*Brevibacterium*, *Lactobacillus*, *Lactococcus*, *Macrococcus*, *Staphylococcus*, *Streptococcus*, and *Escherichia*) that have been detected both on teat skin and in dairy products by other authors^15,32^. The overwhelming majority of OTUs detected in raw milk (85%) were also found on teat skin of cows, although their abundance rank could be very different depending on the habitat. In our study, teats were treated with a post-dipping solution devoid of antiseptic activity and promoting regeneration of the lipid layer^32^. This treatment may help preserving microbial diversity on teat skin, compared to iodinated products with disinfecting action commonly used in dairy farms, and it could promote the transfer of useful microorganisms from teat skin to raw milk. We noticed the presence of technologically important bacteria, such as *Lactococcus*, *Lactobacillus*, and *Streptococcus*, both on teat skin and in milk. Our results confirm that the teat skin is a major reservoir of microorganisms for raw milk^6^. We further showed that the teat skin of cows functions as a secondary reservoir of microorganisms for raw milk cheeses. Approximately a quarter of OTUs detected in cheeses (27%) were also found on teat skin of cows. Several OTUs were assigned to species involved in flavour, aroma and colour development in cheese such as *Brevibacterium linens* and *Staphylococcus equorum*. Other OTUs belonging to lactic acid bacteria such as *Lc*. *lactis*, *Lc*. *chungangsensis/raffinolatis* and *Lb*. *casei/paracasei* could contribute to protein and fat metabolism^27,29^.

A small proportion of OTUs (17 OTUs on 112) were detected in raw milk but not on teat skin. We can hypothesise that these OTUs originated from the milking machine, the milking parlour environment and/or the cheese-making vat^12,33^. The presence of OTUs assigned to the phylum *Proteobacteria* might be a consequence of storage temperature and storage duration during the transport of the milk from the dairy farm to the cheese-making facility^13,34^. Approximately 63% of OTUs found on cheese rind were not detected either on teat skin or in milk in agreement with Wolf *et al*.^35^. The low amount of DNA in raw milk could explain the lower diversity coverage, as described by the rarefaction curves (Supplementary Fig. S4), and the greater variability of observed OTUs numbers and Chao1 in milk than in the other habitats. Therefore the possibility cannot be excluded that OTUs shared with cheeses were present in milk below the detection threshold. However, unlike the cheese core, the rind is an open ecosystem in contact with the external environment^16^. Bokulich and Mills^12^ showed that bacteria and fungi in the environment of cheese-making and ripening cellar (cheese-making equipment, shelves, human contamination, air, water) can also be present in cheeses. Even more so than these environmental microorganisms, the starter and ripening cultures added to milks might compete with the “house” milk microbiota in cheeses. This could explain the shift in dominant OTU composition and the low number of co-occurring OTUs between milk and cheese.

## Conclusion

In this experiment, we aimed to explore whether the bacterial community assembly from the teat skin of cows through to ripened cheeses may be modified by the grazing systems. We highlighted that more than three-quarters of the OTUs detected on teat skin were found in milk, and at least one quarter in cheese. For the first time, our results disclose the important role of cow teat skin as a potential secondary source for microbial diversity in cheese. In addition, the grazing system appeared to be a key driver of cow teat skin microbiota assembly. Overall, this study offers new perspectives on the teat microbiota as a potential vector for a link to terroir through grazing. However, the impact of grazing systems on milk and cheese microbiota structure was much lower. We hypothesize that in addition to the shared milking, cheese-making, and ripening facilities used in this study (which are likely to have enriched the microbiota of the milk and cheese), the starter and ripening cultures contributed to concealing the effect of grazing systems. The differences could be maximised with more contrasted feeding systems (e.g. outdoor vs. indoor) and a lower dose of starter cultures. Further research is needed on environmental reservoirs such as the phyllosphere and soil surface to better understand microbial community assembly from cow teat skin to cheeses.

## Experimental procedures

All experimental protocols involving live animals and all methods were performed in accordance with the relevant guidelines and regulations.

### Experimental design

The experiment was carried out at the experimental farm of Marcenat (INRA, UE1414 Herbipôle, France), which is located in an upland mountainous grassland area in central France (altitude 1135–1215 m). The study was conducted in 2012 during the 2^nd^ year of a long-term experiment^36^ where a total of 24 Holstein and 24 Montbeliarde spring-calving dairy cows and their corresponding heifers were divided in two independent farmlets denominated extensive (EXT) and semi-extensive (SEMI)^22^. Briefly, the EXT system consisted of 60 ha of highly diversified grasslands fertilized with 30 kg/ha/year of organic nitrogen and no mineral fertilization. Dairy cows received no concentrate. The SEMI system comprised 30 ha of less-diversified grasslands fertilized with 50 kg/ha of organic nitrogen and 27 kg/ha of mineral ammonium nitrates on the paddocks to be grazed. The SEMI dairy cows received daily four kilograms of a pelleted concentrate (40% wheat, 30% barley, 25% maize and 5% molasses) distributed in a collective feed trough. Two periods of three dates each (5^th^, 10^th^, and 12^th^ July 2012; 6^th^, 11^th^, and 13^th^ September 2012) were chosen to examine the effects of the two grazing systems at different phenological stages of grass from pastures on the microbiota of teat skin, milk and cheese. On each date, SEMI and EXT cows groups were milked successively on the same milking machine. All teats of all cows were washed with soapy water using individual wipes before each milking and they received a sterile 85%–glycerol post-dipping solution without any disinfectant at the end of each milking.

### Teat skin swab samples

The day before cheese-making, for each of the 48 cows, skin of all four teats was swabbed prior to the evening milking with a moist sterile swab^1^. Individual swab suspensions were stored at −20 °C until analysed. On the day of analysis, they were thawed at 25 °C then were pooled by grazing system (EXT and SEMI) and by day of cheese-making for metagenomic analyses. Overall, 12 teat swab samples were analysed (2 grazing systems × 2 periods × 3 cheese-making dates).

### Cheese production

For each grazing system and for each cheese-making date in July and in September, the evening milk was cooled to 4 °C, then the next day, the morning milk was added. This bulk milk was carried up to the cheese-making experimental facility (INRA, UMR545, Aurillac) (1h30 transport) in airtight cans filled to the brim. Small-size Cantal cheeses (10 kg) were manufactured from 110 L of EXT or SEMI raw milk in separate vats^22^. All milks were inoculated with 0.05 g 100 kg^−1^ of a mesophilic starter culture (Flora Danica Direct, Chr. Hansen, Arpajon, France, consisting of *Lactococcus lactis* ssp. *lactis*, *Lactococcus lactis* ssp. *cremoris*, *Lactococcus lactis* ssp. *lactis biovar diacetylactis* and *Leuconostoc mesenteroides* spp. *cremoris*), then a ripening starter (1.3 mL of Monilev consisting of *Debaryomyces hansenii* and *Sporendonema casei* and 1.3 mL of Penbac consisting of *Brachybacterium tyrofermentans*, *Brevibacterium linens* and *Penicillium fuscoglaucum*, Laboratoire Interprofessionnel de Production, Aurillac, France) was inoculated on hessian cloths at the stage of moulding. Overall, 12 cheeses made from raw milk (2 grazing systems × 2 periods × 3 cheese-making dates) were ripened during 20 weeks. No difference in cheese-making technological parameters and cheese gross biochemical characteristics was observed between the three replicates in each condition^22^. Milk and cheese samples were collected and stored^22^ until the bacterial population counts and metabarcoding analyses.

### Diversity of cultivable bacteria in milks

Milk samples collected before the addition of rennet and starter were enumerated on four elective media for the main microbial groups of milk: total mesophilic bacteria on plate count agar medium (PCAM), lactic acid bacteria on De Man Rogosa Sharpe medium (MRS), Gram-positive catalase-positive bacteria on cheese-ripening bacterial medium (CRBM) and Gram-negative bacteria on PCAM with vancomycin and crystal violet added (PCAI)^22^. For each milk sample, one specimen of each different colony morphotype was picked up from each culture medium and mixed with 2 µL of sterile water. PCR amplifications of 16S rRNA genes (~1500 bp) were carried out directly from 2 µL of water suspensions using the universal primers W02 (5′-GNTACCTTGTTACGACTT-3′) and W18 (5′-AGAGTTTGATCMTGGCTCAG-3′). Each amplification reaction consisted of 50 µL (total volume) and contained 4 µL of a deoxynucleoside triphosphate mixture (dNTP; 2.5 mM), 5 µL of 10X buffer with MgCl_2_, 0.4 µL of Taq DNA polymerase (5 U), and 1.5 µL of each primer (10 µM). The PCR amplification was performed under the following conditions: 10 min at 94 °C, 30 cycles of amplification of 30 s at 94 °C, 30 s at 50 °C and 1 min 30 s at 72 °C, followed by a final extension step of 72 °C for 10 min. The 253 amplified products (112 colonies from PCAM, 17 from MRS, 51 from CRBM, and 73 from PCAi) were sequenced using the W18 primer by LGC Genomics GmbH (Berlin, Germany). The 800 bp of the 5′ ends obtained for the 16S rRNA genes of the isolates were compared to sequences available in the GenBank database, using the BLASTn program. Sequences with a percentage similarity of 99% or higher were considered to be representative of the same species.

### Metabarcoding analysis

#### Phenol-based extraction and purification of total bacterial DNA

The total DNA extraction from core and rind of cheeses (500 mg) was performed as previously described by^37^, including mechanical lysis followed by phenol-based extraction. To extract DNA from teat skin swabs and raw milk, 150 µL of pronase (10 mg/mL; Merck KGaA, Darmstadt, Germany) were added to 10 mL of teat swab suspension and milk samples and heated at 37 °C for 2h30. One milliliter of SDS (sodium dodecyl sulphate 20% solution) was added to each sample, which was then incubated for 1 h at 37 °C. The fat layer was extracted after 20 min of centrifugation (8500 g, 4 °C). The following steps were as described by Duthoit *et al*.^37^. At the end, the nucleic acids pellet was washed, dried, suspended in 50 µL (teat skin swabs and raw milk) or 70 µL (cheese) of water and treated with 5 µL (or 7 µL) of RNase (10 µg/mL; VWR International S.A.S., Fontenay-sous-Bois, France). All the DNA solutions (teat swab suspension, milk, and cheese) were stored at −20 °C.

#### Next generation amplicon sequencing

The total DNA extracted from teat suspension and milk samples (extracted DNA) was amplified using the Illustra^TM^ Ready-To-Go^TM^ GenomiPhi^TM^ V3 DNA Amplification Kit (GE Healthcare, Buckinghamshire, UK).

The variable region V3-V4 of the 16S rRNA gene (~510 bp) was directly amplified from 2 µL of extracted and pre-amplified DNA (teat swab suspension and milk) or extracted DNA (cheese) with the primers PCR1F_460 (5′-TACGGRAGGCAGCAG-3′) and PCR1R_460 (5′-TTACCAGGGTATCTAATCCT-3′) using the following protocol: 94 °C for 60 s; 30 cycles of 94 °C for 60 s, 65 °C for 60 s, 72 °C for 60 s, and 72 °C for 10 min. The amplification reaction was carried out in a final volume of 50 µL containing 1 µL of dNTP mixture (10 mM), 1.25 µL of each primer (20 µM), 5 µL of 10X MTP Taq Buffer and 0.5 µL of 5 U MTP^TM^ Taq DNA polymerase (Sigma-Aldrich, France). The amplified products were used for Illumina paired-end library preparation and cluster generation (INRA, UMR1388 GenPhySe, France), followed by 250 bp paired-end sequencing on an Illumina MiSeq instrument (INRA, GeT-PLaGE plateform, Toulouse, France). Raw sequence data were deposited at the Sequence Read Archive of the National Center for Biotechnology Information under the accession numbers SRR6365131 to SRR6365167.

#### Data analysis

The sequence data were processed using FROGS pipeline on Galaxy interface^38^. After a quality control, the contiged reads were filtered to minimize the effects of random sequencing errors: (i) contigs with a length between 400 and 500 bp were kept, (ii) contigs where the two primers were missing were eliminated, (ii) contigs with ambiguous nucleotides were discarded. Reads were clustered (aggregation distance of 3) with Swarm^39^, then chimeric sequences in each sample were removed using VSEARCH tool^40^. To filter on singletons (OTU with a single read) and chimeric sequences, OTUs with proportional counts below 0.005% across all samples were removed^41^. The taxonomy assignment was performed with both NCBI blastn+ and RDP Classifier against the pre-processed SILVA123-16S database. In order to analyse the effect of grazing system in each habitat (teat skin, milk, cheese core or rind), the OTUs present in a single sample among all samples from a given habitat were removed.

### Statistical analyses

Rarefaction curves, diversity indices of Shannon^42^, Simpson^43^, and richness estimator Chao^44^ were calculated on non-normalised data to infer on richness and evenness in the samples. These data were analysed by a multi-way analysis of variance (ANOVA) with R (version 3.1.0), including variables corresponding to the grazing system used, the period of cheese-making, the habitat and their interactions as fixed factors. Significance was declared at *P* < 0.05. Next, count data for each individual sample were divided by the ratio between the total sum of the filtered reads of the sample and the total sum of the sample of each given habitat (teat swab, cheese core or cheese rind) with the lowest sequencing depth. For milk samples, the rarefaction curves didn’t show an asymptote suggesting an insufficient sampling of the bacterial community: this step of normalisation could not be performed (Supplementary Fig. S4). The linear discriminant analysis (LDA) effect size (LEfSe) method (available at http://huttenhower.sph.harvard.edu/galaxy) was used with the relative abundance data to ascertain any significant differences in taxonomic abundance between two grazing systems^45^. The LDA analysis generates a list of discriminant taxonomic units with a *P* < 0.05. The LDA score allows to rank the OTUs based on their relevance, between 5.2 and 2.5 with the present dataset. The multivariate partial least squares discriminant analysis (PLS-DA) was performed on the normalized log-transformed OTU abundance of overall samples considering as factor the combination of qualitative variables: habitats (cow teat skin, milk, cheese core and cheese rind) and grazing systems (EXT and SEMI). PLS-DA was carried out with the R package mixOmics^46^. The OTU networks were generated by QIIME pipeline^47^ and networks were visualized using Cytoscape 3.4.0^48^ by applying the spring embedded layout. Venn diagrams were generated using Jvenn (available at http://bioinfo.genotoul.fr/jvenn) ^49^.

### Ethical approval

All prevailing local, national and international regulations and conventions, and normal scientific ethical practices, have been respected. All methods were performed in accordance with the relevant guidelines and regulations. All experimental protocols involving live animals were approved by the institutional Ethics Committee in Animal Experiment, CEMEA Auvergne (authorization N°CE 21-13).

## Electronic supplementary material

Frétin et al. Supplementary Tables and Figures
