# Supplementary material for: Bacterial community assembly from cow teat skin to ripened cheeses is influenced by grazing systems
Source: Sci Rep. 2018 Jan 9;8:200. doi: 10.1038/s41598-017-18447-y (PMC5760519; doi:10.1038/s41598-017-18447-y)
Supplement: Supplementary file 1 — Frétin et al. Supplementary Tables and Figures [file 41598_2017_18447_MOESM1_ESM.pdf]

# Bacterial community assembly from cow teat skin to ripened cheeses is influenced by grazing systems

Frétin Marie[1,2], Martin Bruno[2], Rifa Etienne[1], Verdier-Metz Isabelle[1], Pomiès Dominique[2], Ferlay Anne[2], Montel Marie-Christine[1], Delbès Céline[1*]

**Table S1.** Overall bacterial composition in teat skin and dairy product samples

| | Teat skin (*n=12*) | Raw milk (*n=12*) | Cheese core (*n=12*) | Cheese rind (*n=12*) |
|---|---|---|---|---|
| Total OTU | 300 | 112 | 43 | 75 |
| Total number of genera | 98 | 52 | 14 | 23 |
| Total OTUs > 1% | 18 | 17 | 1 | 5 |

*n*: number of samples by habitat

**Table S2.** Taxonomic distribution of isolates obtained from raw milk

| Phylum | Genus and/or species[1] | July | | September | |
|---|---|---|---|---|---|
| | | EXT[2] | SEMI[3] | EXT[2] | SEMI[3] |
| *Actinobacteria* (32.4%) | *Bifidobacterium crudilactis* | 2 | 1 | 1 | |
| | *Gordonia sputi* | | | | 1 |
| | *Rhodococcus coprophilus* | | | | 1 |
| | *Salana* sp. | 1 | | | |
| | *Brachybacterium paraconglomeratum* | 1 | | 1 | 1 |
| | *Dermacoccus nishinomiyaensis* | | 1 | 1 | 1 |
| | *Curtobacterium flaccumfaciens* | | | | 1 |
| | *Leifsonia aquatica* | | | 1 | |
| | *Microbacterium aurum* | | 1 | | |
| | *Microbacterium flavum* | 1 | | 1 | |
| | *Microbacterium lacticum* | | 2 | | 2 |
| | *Microbacterium oxydans* | 5 | 2 | 2 | 1 |
| | *Microbacterium* sp. | | | | 1 |
| | *Pseudoclavibacter helvolus* | | | 2 | |
| | *Kocuria carniphila* | 1 | 1 | | |
| | *Kocuria palustris* | | | 2 | 1 |
| | *Kocuria rhizophila* | 1 | | | |
| | *Kocuria salsicia* | 9 | 8 | 5 | 3 |
| | *Propioniciclava* sp. | 3 | 5 | 4 | 4 |
| | Total for phylum | 24 | 21 | 20 | 17 |
| *Bacteroidetes* (5.5%) | *Chryseobacterium bovis* | 1 | | | 1 |
| | *Chryseobacterium meningosepticum* | 1 | | | |
| | *Chryseobacterium oncorhynchi* | 1 | | | |
| | *Chryseobacterium shigense* | 1 | 4 | | |
| | *Chryseobacterium* sp. | | 1 | 2 | 2 |
| | Total for phylum | 4 | 5 | 2 | 3 |
| *Deinococcus-Thermus* (1.2%) | *Deinococcus* sp. | | | 2 | |
| | *Deinococcus xinjiangensis* | 1 | | | |
| | Total for phylum | 1 | 0 | 2 | 0 |
| *Firmicutes* (33.2%) | *Bacillus cereus* | 2 | | | |
| | *Bacillus pumilus* | | | 1 | |
| | *Bacillus* sp. | | 2 | | |
| | *Macrococcus caseolyticus* | 2 | 2 | | |
| | *Staphylococcus aureus* | 2 | 1 | 1 | 1 |
| | *Staphylococcus chromogenes* | 3 | 2 | 1 | 2 |
| | *Staphylococcus devriesei* | 1 | 3 | 3 | |
| | *Staphylococcus haemolyticus* | 4 | 2 | 5 | 2 |
| | *Staphylococcus saprophyticus* | | 1 | 1 | |
| | *Staphylococcus* sp. | 1 | 1 | | |
| | *Aerococcus viridans* | | | | 1 |
| | *Enterococcus faecium* | | | | 2 |
| | *Enterococcus gilvus* | | | 1 | |
| | *Lactobacillus casei* | | 5 | 5 | 9 |
| | *Lactobacillus helveticus* | 1 | 2 | 3 | 1 |
| | *Lactobacillus parabuchneri* | | 1 | 1 | |
| | *Lactobacillus paracasei* | | 1 | | |
| | *Leuconostoc citreum* | 1 | | | |
| | *Lactococcus lactis* | 2 | 1 | | |

| | | | | | |
|---|---|---|---|---|---|
| | *Streptococcus uberis* | | | | 1 |
| | Total for phylum | 19 | 24 | 22 | 19 |
| *Proteobacteria* (27.7%) | *Brevundimonas aurantiaca* | 2 | | | |
| | *Brevundimonas intermedia* | 3 | | | |
| | *Brevundimonas nasdae* | | 1 | | |
| | *Brevundimonas* sp. | 4 | 4 | 1 | 3 |
| | *Brevundimonas vesicularis* | 2 | 2 | | 1 |
| | *Ochrobactrum rhizosphaerae* | 1 | 1 | | 1 |
| | *Devosia* sp. | | 1 | | |
| | *Rhizobium radiobacter* | | | 1 | |
| | *Rhizobium* sp. | | 1 | | |
| | *Shinella zoogloeoides* | 2 | | | |
| | *Paracoccus yeei* | 1 | | | |
| | *Sphingomonas melonis* | 2 | | | |
| | *Sphingomonas* sp. | | | | 1 |
| | *Variovorax paradoxus* | 1 | | | |
| | *Escherichia coli* | 1 | | | |
| | *Pseudomonas* sp. | | | | 1 |
| | *Stenotrophomonas maltophilia* | 16 | 4 | 1 | |
| | *Stenotrophomonas rhizophila* | | 3 | | |
| | *Stenotrophomonas* sp. | | 2 | 3 | 3 |
| | Total for phylum | 35 | 19 | 6 | 10 |
| Total identified | | 83 | 69 | 52 | 49 |

Values reported correspond to the number of clones identified from triplicate milk sample
[1] The underlined genera were not detected by 16S rRNA gene metabarcoding in raw milks
[2] EXT = extensive grazing system; [3]SEMI = semi-intensive grazing system

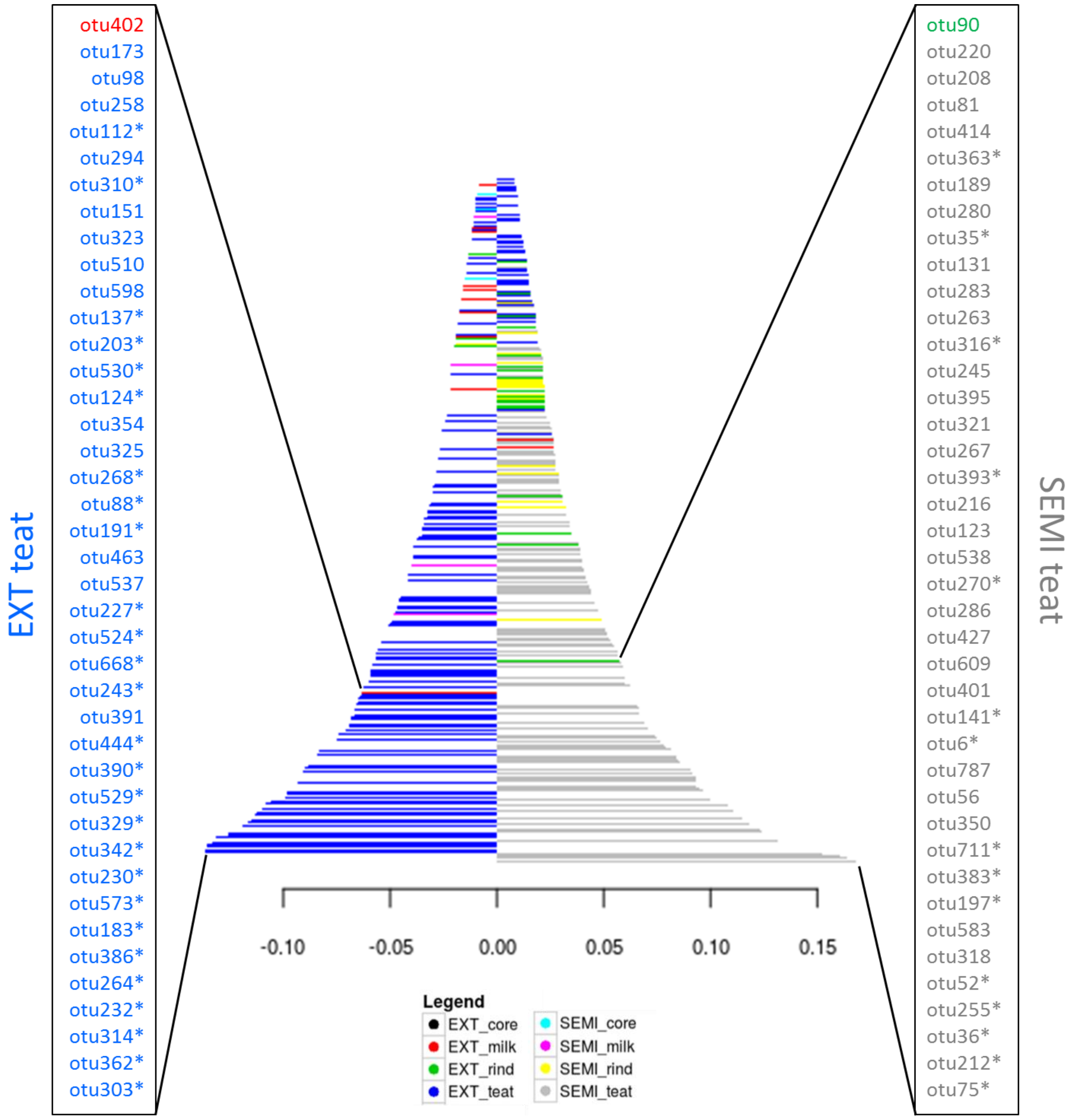


**Figure S1.** Contribution to PC3 of 365 variables of the PLS-DA performed on all the samples (teat skin swab, milk, cheese core and rind) of July. The 82 OTUs that contribute the most to PC3 were reported. The symbol * indicates the OTUs with a differential abundance using LEfSe (Table S2 and S3).

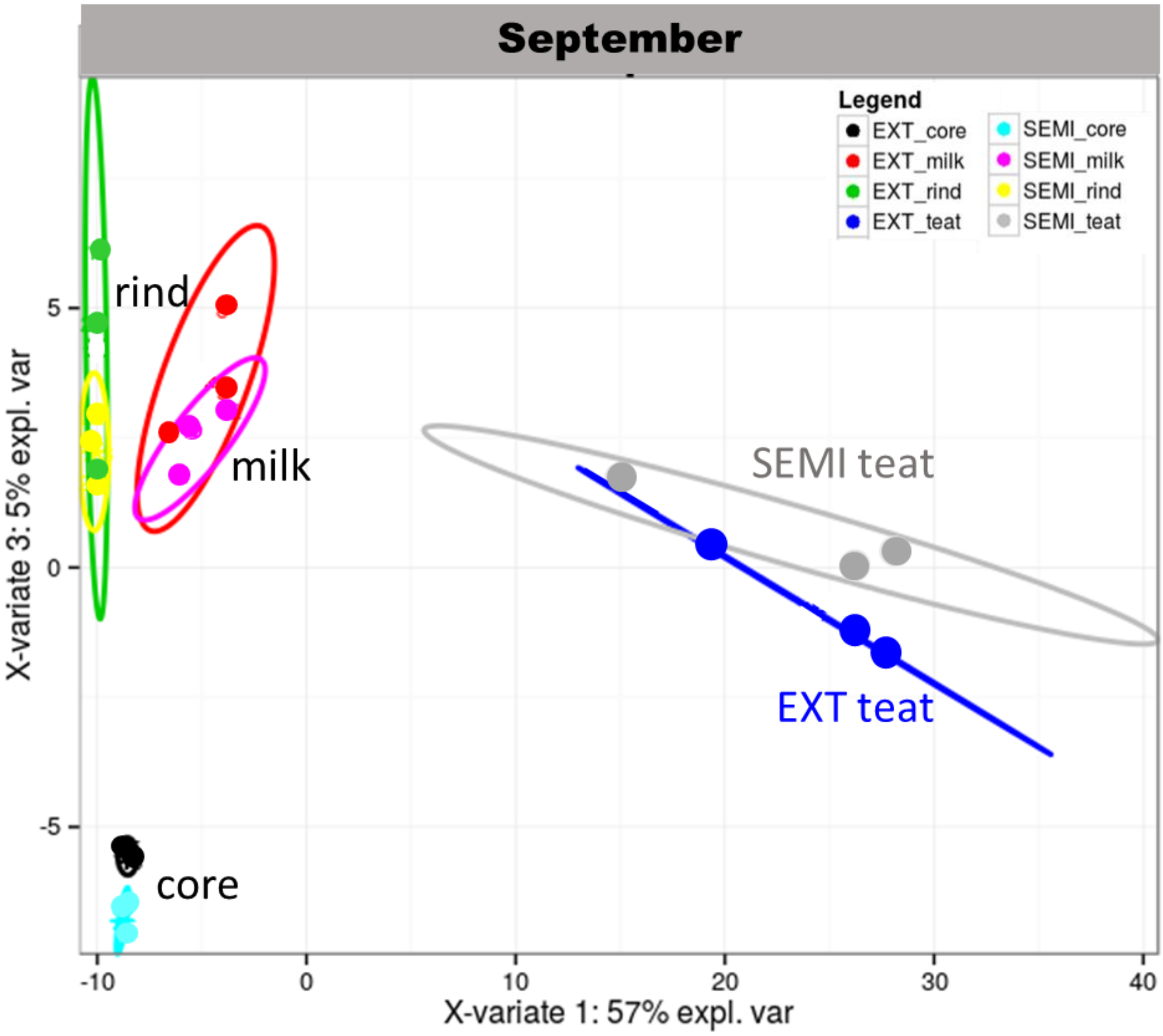


**Figure S2.** PLS-DA performed on the relative abundance of 365 OTUs found on all the samples of September from two grazing systems. Plot of sample distribution is projected on principal components 1 and 3.

**Table S3.** Differentially abundant taxa on teat skin in July between EXT (*n*=3) and SEMI (*n*=3) systems as determined by the LDA method[1].

| Notation on Fig. 2A | Taxa | N°OTU | Average abundance | | Standard deviation | | LDA score | pval |
|---|---|---|---|---|---|---|---|---|
| | | | EXT | SEMI | EXT | SEMI | | |
| Taxa more abundant in EXT | | | | | | | | |
| | ***Actinobacteria*** | | | | | | | |
| f | Actinobacteria\|Corynebacteriales\|Corynebacteriaceae\|Corynebacterium\|Corynebacterium spp. | OTU12 | 3.95 | 0.96 | 2.23 | 0.26 | 4.166 | 0.0495 |
| g | Actinobacteria\|Corynebacteriales\|Corynebacteriaceae\|Corynebacterium | **OTU137** | 0.27 | 0.02 | 0.18 | 0.02 | 3.119 | 0.0495 |
| h | Actinobacteria\|Corynebacteriales\|Corynebacteriaceae\|Corynebacterium | **OTU183** | 0.19 | - | 0.24 | - | 3.096 | 0.0369 |
| i | Actinobacteria\|Corynebacteriales\|Corynebacteriaceae\|Corynebacterium | **OTU444** | 0.09 | <0.01 | 0.06 | 0.00 | 2.713 | 0.0463 |
| | Coriobacteriia | class | 1.72 | 0.60 | 0.66 | 0.17 | 4.242 | 0.0495 |
| | Coriobacteriia\|Coriobacteriales | order | 1.72 | 0.60 | 0.66 | 0.17 | 4.198 | 0.0495 |
| r | Coriobacteriia\|Coriobacteriales\|Coriobacteriaceae | family | 1.72 | 0.60 | 0.66 | 0.17 | 3.763 | 0.0495 |
| k | Coriobacteriia\|Coriobacteriales\|Coriobacteriaceae\|Atopobium | genus | 1.53 | 0.46 | 0.70 | 0.15 | 3.742 | 0.0495 |
| l | Coriobacteriia\|Coriobacteriales\|Coriobacteriaceae\|Atopobium | **OTU112** | 0.67 | 0.05 | 0.36 | 0.03 | 3.512 | 0.0495 |
| m | Coriobacteriia\|Coriobacteriales\|Coriobacteriaceae\|Atopobium | **OTU124** | 0.36 | 0.05 | 0.19 | 0.07 | 3.207 | 0.0495 |
| n | Coriobacteriia\|Coriobacteriales\|Coriobacteriaceae\|Atopobium | OTU130 | 0.35 | 0.11 | 0.23 | 0.04 | 3.096 | 0.0495 |
| q | Coriobacteriia\|Coriobacteriales\|Coriobacteriaceae\|Gordonibacter | **OTU329** | 0.07 | 0.01 | 0.03 | 0.01 | 2.945 | 0.0495 |
| | ***Firmicutes*** | | | | | | | |
| u | Bacilli\|Bacillales\|Planococcaceae\|Solibacillus | OTU205 | 0.10 | 0.06 | 0.01 | 0.04 | 2.760 | 0.0495 |
| x | Bacilli\|Lactobacillales\|Aerococcaceae\|Aerococcus | genus | 2.39 | 0.41 | 2.54 | 0.19 | 3.982 | 0.0495 |
| y | Clostridia\|Clostridiales\|Christensenellaceae | OTU146 | 0.50 | 0.07 | 0.22 | 0.06 | 3.366 | 0.0495 |
| z | Clostridia\|Clostridiales\|Christensenellaceae | OTU193 | 0.10 | 0.02 | 0.03 | 0.01 | 2.797 | 0.0495 |
| a0 | Clostridia\|Clostridiales\|Christensenellaceae | **OTU227** | 0.09 | 0.01 | 0.05 | 0.00 | 2.655 | 0.0495 |
| a1 | Clostridia\|Clostridiales\|Christensenellaceae | OTU258 | 0.21 | 0.03 | 0.13 | 0.03 | 2.959 | 0.0495 |
| a2 | Clostridia\|Clostridiales\|Christensenellaceae | **OTU268** | 0.08 | 0.01 | 0.05 | 0.01 | 2.688 | 0.0495 |
| a3 | Clostridia\|Clostridiales\|Christensenellaceae | OTU285 | 0.28 | 0.03 | 0.23 | 0.02 | 3.119 | 0.0495 |
| a4 | Clostridia\|Clostridiales\|Christensenellaceae | **OTU303** | 0.09 | - | 0.01 | - | 2.962 | 0.0369 |
| a5 | Clostridia\|Clostridiales\|Christensenellaceae | **OTU390** | 0.15 | <0.01 | 0.17 | 0.01 | 2.879 | 0.0463 |
| a6 | Clostridia\|Clostridiales\|Christensenellaceae | OTU398 | 0.06 | 0.02 | 0.02 | 0.00 | 2.936 | 0.0495 |

| | | | | | | | | |
|---|---|---|---|---|---|---|---|---|
| a7 | Clostridia\|Clostridiales\|Christensenellaceae | OTU402 | 0.09 | 0.02 | 0.05 | 0.01 | 2.670 | 0.0495 |
| a8 | Clostridia\|Clostridiales\|Christensenellaceae | **OTU530** | 0.06 | 0.01 | 0.05 | 0.00 | 2.525 | 0.0495 |
| b3 | Clostridia\|Clostridiales\|FamilyXIII | family | 0.30 | 0.07 | 0.16 | 0.04 | 3.108 | 0.0495 |
| b2 | Clostridia\|Clostridiales\|Family XIII\|Mogibacterium | OTU135 | 0.30 | 0.07 | 0.16 | 0.04 | 3.096 | 0.0495 |
| c1 | Clostridia\|Clostridiales\|Lachnospiraceae | family | 4.63 | 1.47 | 2.51 | 0.68 | 4.213 | 0.0495 |
| d1 | Clostridia\|Clostridiales\|Lachnospiraceae | **OTU88** | 0.56 | 0.04 | 0.22 | 0.05 | 3.428 | 0.0495 |
| c2 | Clostridia\|Clostridiales\|Lachnospiraceae | OTU156 | 0.15 | 0.02 | 0.12 | 0.01 | 2.859 | 0.0495 |
| c3 | Clostridia\|Clostridiales\|Lachnospiraceae | OTU181 | 0.13 | 0.04 | 0.04 | 0.02 | 2.743 | 0.0495 |
| c4 | Clostridia\|Clostridiales\|Lachnospiraceae | **OTU203** | 0.27 | 0.03 | 0.16 | 0.01 | 3.106 | 0.0495 |
| c5 | Clostridia\|Clostridiales\|Lachnospiraceae | **OTU232** | 0.04 | - | 0.01 | - | 2.951 | 0.0369 |
| c6 | Clostridia\|Clostridiales\|Lachnospiraceae | **OTU243** | 0.10 | 0.01 | 0.05 | 0.01 | 2.730 | 0.0495 |
| c8 | Clostridia\|Clostridiales\|Lachnospiraceae | OTU313 | 0.08 | 0.01 | 0.02 | 0.01 | 2.725 | 0.0495 |
| c9 | Clostridia\|Clostridiales\|Lachnospiraceae | **OTU362** | 0.08 | <0.01 | 0.00 | 0.00 | 2.945 | 0.0463 |
| d0 | Clostridia\|Clostridiales\|Lachnospiraceae | **OTU529** | 0.05 | <0.01 | 0.02 | 0.01 | 2.858 | 0.0495 |
| b4 | Clostridia\|Clostridiales\|Lachnospiraceae\|Acetitomaculum | | 1.25 | 0.11 | 0.65 | 0.04 | 3.796 | 0.0495 |
| b7 | Clostridia\|Clostridiales\|Lachnospiraceae\|Acetitomaculum | OTU37 | 0.97 | 0.08 | 0.56 | 0.04 | 3.637 | 0.0495 |
| b5 | Clostridia\|Clostridiales\|Lachnospiraceae\|Acetitomaculum | **OTU264** | 0.09 | <0.01 | 0.04 | 0.00 | 2.725 | 0.0463 |
| b6 | Clostridia\|Clostridiales\|Lachnospiraceae\|Acetitomaculum | **OTU314** | 0.07 | <0.01 | 0.01 | 0.00 | 2.863 | 0.0463 |
| b8 | Clostridia\|Clostridiales\|Lachnospiraceae\|Acetitomaculum | **OTU573** | 0.05 | - | 0.03 | - | 2.529 | 0.0369 |
| c0 | Clostridia\|Clostridiales\|Lachnospiraceae\|Coprococcus | **OTU191** | 0.19 | 0.02 | 0.08 | 0.02 | 2.984 | 0.0495 |
| d5 | Clostridia\|Clostridiales\|Ruminococcaceae | **OTU230** | 0.09 | <0.01 | 0.01 | 0.01 | 2.991 | 0.0463 |
| d6 | Clostridia\|Clostridiales\|Ruminococcaceae | **OTU310** | 0.13 | 0.02 | 0.11 | 0.01 | 2.784 | 0.0495 |
| d7 | Clostridia\|Clostridiales\|Ruminococcaceae | **OTU342** | 0.09 | <0.01 | 0.04 | 0.01 | 2.727 | 0.0463 |
| d9 | Clostridia\|Clostridiales\|Ruminococcaceae | **OTU668** | 0.07 | <0.01 | 0.08 | 0.01 | 2.594 | 0.0463 |
| d3 | Clostridia\|Clostridiales\|Ruminococcaceae\|Ruminiclostridium | **OTU524** | 0.09 | 0.01 | 0.09 | 0.01 | 2.748 | 0.0463 |
| d4 | Clostridia\|Clostridiales\|Ruminococcaceae\|Saccharofermentans | OTU356 | 0.16 | 0.04 | 0.04 | 0.00 | 2.871 | 0.0495 |
| e0 | Erysipelotrichia\|Erysipelotrichales\|Erysipelotrichaceae\|Solobacterium | **OTU386** | 0.05 | <0.01 | 0.03 | 0.00 | 2.709 | 0.0463 |
| Taxa more abundant in SEMI | | | | | | | | |
| | ***Actinobacteria*** | | | | | | | |
| | Actinobacteria\|Bifidobacteriales | order | 0.48 | 10.5 | 0.59 | 5.69 | 4.694 | 0.0495 |
| e | Actinobacteria\|Bifidobacteriales\|Bifidobacteriaceae | family | 0.48 | 10.5 | 0.59 | 5.69 | 4.675 | 0.0495 |

| | | | | | | | | |
|---|---|---|---|---|---|---|---|---|
| d | Actinobacteria\|Bifidobacteriales\|Bifidobacteriaceae\|Bifidobacterium | genus | 0.48 | 10.5 | 0.59 | 5.69 | 4.674 | 0.0495 |
| c | Actinobacteria\|Bifidobacteriales\|Bifidobacteriaceae\|Bifidobacterium\|B.pseudolongum | **OTU6** | 0.46 | 9.90 | 0.58 | 5.46 | 4.640 | 0.0495 |
| a | Actinobacteria\|Bifidobacteriales\|Bifidobacteriaceae\|Bifidobacterium\|B.choerinum | **OTU36** | <0.01 | 0.34 | 0.00 | 0.30 | 3.238 | 0.0495 |
| b | Actinobacteria\|Bifidobacteriales\|Bifidobacteriaceae\|Bifidobacterium\|B.merycicum | **OTU52** | 0.02 | 0.27 | 0.01 | 0.07 | 3.118 | 0.0495 |
| j | Actinobacteria\|Propionibacteriales\|Nocardioidaceae | **OTU363** | 0.02 | 0.06 | 0.00 | 0.07 | 3.107 | 0.0495 |
| p | Coriobacteriia\|Coriobacteriales\|Coriobacteriaceae\|Atopobium | **OTU75** | - | 0.15 | - | 0.02 | 3.033 | 0.0369 |
| o | Coriobacteriia\|Coriobacteriales\|Coriobacteriaceae\|Atopobium | **OTU212** | - | 0.04 | - | 0.02 | 3.026 | 0.0369 |
| | ***Firmicutes*** | | | | | | | |
| s | Bacilli\|Bacillales\|Bacillaceae\|Bacillus | **OTU141** | 0.06 | 0.49 | 0.03 | 0.33 | 3.362 | 0.0495 |
| t | Bacilli\|Bacillales\|Paenibacillaceae\|Paenibacillus\|Paenibacillus spp. | OTU359 | 0.05 | 0.08 | 0.02 | 0.00 | 2.676 | 0.0495 |
| w | Bacilli\|Bacillales\|Planococcaceae\|Sporosarcina | **OTU393** | 0.02 | 0.06 | 0.01 | 0.01 | 2.891 | 0.0495 |
| v | Bacilli\|Bacillales\|Planococcaceae\|Sporosarcina\|S. globispora | **OTU316** | 0.03 | 0.07 | 0.01 | 0.01 | 2.666 | 0.0495 |
| a9 | Clostridia\|Clostridiales\|Clostridiaceae\|Clostridium | genus | 0.62 | 2.35 | 0.26 | 0.91 | 3.989 | 0.0495 |
| b0 | Clostridia\|Clostridiales\|Clostridiaceae\|Clostridium\|Clostridium spp. | **OTU35** | 0.34 | 1.89 | 0.12 | 0.83 | 3.881 | 0.0495 |
| b1 | Clostridia\|Clostridiales\|Clostridiaceae\|Clostridium | OTU85 | 0.19 | 0.40 | 0.12 | 0.08 | 3.032 | 0.0495 |
| c7 | Clostridia\|Clostridiales\|Lachnospiraceae | **OTU255** | - | 0.23 | - | 0.22 | 3.134 | 0.0369 |
| b9 | Clostridia\|Clostridiales\|Lachnospiraceae\|Cellulosilyticum | **OTU270** | 0.03 | 0.11 | 0.02 | 0.06 | 2.758 | 0.0495 |
| d2 | Clostridia\|Clostridiales\|Ruminococcaceae | **OTU197** | 0.01 | 0.27 | 0.01 | 0.23 | 3.168 | 0.0463 |
| d8 | Clostridia\|Clostridiales\|Ruminococcaceae | **OTU383** | 0.02 | 0.12 | 0.02 | 0.09 | 2.831 | 0.0495 |
| e1 | Erysipelotrichia\|Erysipelotrichales\|Erysipelotrichaceae\|Turicibacter | **OTU711** | 0.01 | 0.08 | 0.00 | 0.03 | 2.871 | 0.0495 |

[1]Average abundances (expressed as percentages) as well as standard deviation are presented for each taxon. EXT = extensive grazing system; SEMI = semi-intensive grazing system

The bold OTUs correspond to the OTUs that contribute the most to the third principal component (PC3) of PLS-DA on Figure S1.

**Table S4.** Differentially abundant taxa on teat skin in September between EXT ($n$=3) and SEMI ($n$=3) systems as determined by the LDA method[1].

| Notation on Fig. 2B | Taxa | N°OTU | Average abundance | | Standard deviation | | LDA score | pval |
|---|---|---|---|---|---|---|---|---|
| | | | EXT | SEMI | EXT | SEMI | | |
| Taxa more abundant in EXT | | | | | | | | |
| | ***Actinobacteria*** | | | | | | | |
| | Actinobacteria\|Corynebacteriales | order | 34.5 | 17.1 | 9.23 | 5.22 | 4.961 | 0.0495 |
| h | Actinobacteria\|Corynebacteriales\|Corynebacteriaceae | family | 31.9 | 15.5 | 7.49 | 4.75 | 5.172 | 0.0495 |
| f | Actinobacteria\|Corynebacteriales\|Corynebacteriaceae\|Corynebacterium | genus | 31.9 | 15.5 | 7.49 | 4.75 | 4.976 | 0.0495 |
| g | Actinobacteria\|Corynebacteriales\|Corynebacteriaceae\|Corynebacterium | OTU260 | 0.36 | 0.03 | 0.30 | 0.01 | 3.309 | 0.0495 |
| q | Coriobacteriia\|Coriobacteriales\|Coriobacteriaceae\|Gordonibacter | OTU329 | 0.06 | 0.01 | 0.05 | 0.01 | 3.331 | 0.0495 |
| | ***Firmicutes*** | | | | | | | |
| u | Clostridia\|Clostridiales\|Christensenellaceae | OTU370 | 0.05 | 0.01 | 0.03 | 0.01 | 3.402 | 0.0495 |
| a0 | Clostridia\|Clostridiales\|Lachnospiraceae | OTU362 | 0.08 | 0.01 | 0.06 | 0.01 | 3.185 | 0.0495 |
| a1 | Clostridia\|Clostridiales\|Peptostreptococcaceae\|Terrisporobacter | OTU357 | 0.07 | <0.01 | 0.04 | 0.00 | 3.409 | 0.0463 |
| | ***Proteobacteria*** | | | | | | | |
| a6 | Alphaproteobacteria\|Rhizobiales\|Phyllobacteriaceae\|Aminobacter | OTU169 | 0.21 | 0.05 | 0.15 | 0.02 | 3.282 | 0.0495 |
| Taxa more abundant in SEMI | | | | | | | | |
| | ***Actinobacteria*** | | | | | | | |
| | Actinobacteria\|Bifidobacteriales | order | 1.55 | 21.6 | 0.57 | 24.89 | 4.990 | 0.0495 |
| e | Actinobacteria\|Bifidobacteriales\|Bifidobacteriaceae | family | 1.55 | 21.6 | 0.57 | 24.89 | 4.983 | 0.0495 |
| d | Actinobacteria\|Bifidobacteriales\|Bifidobacteriaceae\|Bifidobacterium | genus | 1.55 | 21.6 | 0.57 | 24.89 | 5.021 | 0.0495 |
| c | Actinobacteria\|Bifidobacteriales\|Bifidobacteriaceae\|Bifidobacterium\|B.pseudolongum | OTU6 | 1.36 | 17.5 | 0.62 | 20.96 | 4.904 | 0.0495 |
| a | Actinobacteria\|Bifidobacteriales\|Bifidobacteriaceae\|Bifidobacterium\|B.choerinum | OTU36 | 0.07 | 2.69 | 0.06 | 3.28 | 4.102 | 0.0495 |
| b | Actinobacteria\|Bifidobacteriales\|Bifidobacteriaceae\|Bifidobacterium\|B.merycicum | OTU52 | 0.12 | 1.35 | 0.13 | 0.74 | 3.779 | 0.0495 |
| j | Actinobacteria\|Corynebacteriales\|Nocardiaceae | family | 0.21 | 0.31 | 0.05 | 0.03 | 3.073 | 0.0495 |
| i | Actinobacteria\|Corynebacteriales\|Nocardiaceae\|Rhodococcus | genus | 0.18 | 0.30 | 0.03 | 0.03 | 3.385 | 0.0495 |
| k | Actinobacteria\|Micrococcales\|Dermatophilaceae | OTU281 | 0.03 | 0.05 | 0.02 | 0.01 | 3.380 | 0.0495 |

| | | | | | | | | |
|---|---|---|---|---|---|---|---|---|
| l | Actinobacteria\|Micrococcales\|Microbacteriaceae\|Agreia | OTU335 | 0.01 | 0.04 | 0.00 | 0.02 | 3.424 | 0.0495 |
| | Actinobacteria\|Micromonosporales | Order | 0.01 | 0.03 | 0.01 | 0.00 | 3.781 | 0.0495 |
| n | Actinobacteria\|Micromonosporales\|Micromonosporaceae | family | 0.01 | 0.03 | 0.01 | 0.00 | 3.683 | 0.0495 |
| m | Actinobacteria\|Micromonosporales\|Micromonosporaceae\|Micromonospora | OTU379 | 0.01 | 0.03 | 0.01 | 0.00 | 3.810 | 0.0495 |
| p | Coriobacteriia\|Coriobacteriales\|Coriobacteriaceae\|Atopobium | OTU75 | 0.05 | 1.15 | 0.06 | 0.78 | 3.758 | 0.0495 |
| o | Coriobacteriia\|Coriobacteriales\|Coriobacteriaceae\|Atopobium | OTU212 | <0.01 | 0.28 | 0.00 | 0.36 | 3.445 | 0.0463 |
| | ***Firmicutes*** | | | | | | | |
| r | Bacilli\|Bacillales\|Planococcaceae | OTU302 | 0.01 | 0.13 | 0.02 | 0.08 | 3.017 | 0.0495 |
| t | Bacilli\|Lactobacillales\|Streptococcaceae\|Lactococcus | genus | 0.18 | 0.23 | 0.02 | 0.04 | 3.000 | 0.0495 |
| s | Bacilli\|Lactobacillales\|Streptococcaceae\|Lactococcus\|Lc. lactis | OTU1 | 0.18 | 0.21 | 0.01 | 0.02 | 3.566 | 0.0495 |
| x | Clostridia\|Clostridiales\|Clostridiaceae\|Clostridium | genus | 0.49 | 1.18 | 0.23 | 0.55 | 3.581 | 0.0495 |
| y | Clostridia\|Clostridiales\|Clostridiaceae\|Clostridium spp. | OTU35 | 0.26 | 0.72 | 0.01 | 0.05 | 3.369 | 0.0495 |
| w | Clostridia\|Clostridiales\|Clostridiaceae\|Clostridium\|C. hylemonae | OTU271 | 0.01 | 0.07 | - | 0.09 | 3.284 | 0.0495 |
| v | Clostridia\|Clostridiales\|Clostridiaceae\|Clostridium\|C. chartababidum | OTU321 | - | 0.11 | 0.23 | 0.55 | 2.996 | 0.0369 |
| z | Clostridia\|Clostridiales\|Lachnospiraceae\|Coprococcus | OTU248 | - | 0.02 | - | 0.03 | 3.260 | 0.0369 |
| a2 | Clostridia\|Clostridiales\|Ruminococcaceae | OTU197 | - | 0.04 | - | 0.03 | 3.217 | 0.0463 |
| a3 | Clostridia\|Clostridiales\|Ruminococcaceae\|Ruminococcus | OTU216 | 0.01 | 0.28 | 0.01 | 0.20 | 3.188 | 0.0495 |
| a4 | Clostridia\|Clostridiales\|Ruminococcaceae\|Ruminococcus | OTU322 | 0.01 | 0.08 | 0.00 | 0.05 | 3.666 | 0.0495 |
| a5 | Clostridia\|Clostridiales\|Ruminococcaceae\|Ruminococcus | OTU334 | 0.02 | 0.11 | 0.01 | 0.11 | 3.131 | 0.0495 |

[1]Average abundances (expressed as percentages) as well as standard deviation are presented for each taxon. EXT = extensive grazing system; SEMI = semi-intensive grazing system

**Table S5.** Differentially abundant taxa in raw milk **(A)** in July and **(B)** in September between EXT ($n$=3) and SEMI ($n$=3) systems as determined by the LDA method[1].

**A**

| Taxa | Average abundance | | Standard deviation | | LDA score | pval |
|---|---|---|---|---|---|---|
| | EXT | SEMI | EXT | SEMI | | |
| Taxa more abundant in EXT | | | | | | |
| ***Firmicutes*** | | | | | | |
| Clostridia\|Clostridiales\|Lachnospiraceae | 0.33 | 0.06 | 0.19 | 0.06 | 3.546 | 0.0495 |
| ***Proteobacteria*** | | | | | | |
| Gammaproteobacteria | 3.48 | 0.14 | 2.98 | 0.08 | 4.308 | 0.0495 |
| Gammaproteobacteria\|Pseudomonadales | 3.09 | 0.01 | 2.47 | 0.02 | 4.234 | 0.0463 |

**B**

| Taxa | Average abundance | | Standard deviation | | LDA score | pval |
|---|---|---|---|---|---|---|
| | EXT | SEMI | EXT | SEMI | | |
| Taxa more abundant in EXT | | | | | | |
| ***Actinobacteria*** | | | | | | |
| Actinobacteria\|Corynebacteriales\|Nocardiaceae\|Rhodococcus\|Rhodococcus spp._otu100 | 3.26 | 0.82 | 1.07 | 1.32 | 4.209 | 0.0495 |
| Actinobacteria\|Micrococcales\|Micrococcaceae\|Kocuria\|Kocuria spp._otu72 | 0.09 | - | 0.12 | - | 4.131 | 0.0339 |
| Taxa more abundant in SEMI | | | | | | |
| ***Firmicutes*** | 19.9 | 48.5 | 6.37 | 18.6 | 5.177 | 0.0495 |
| Clostridia\|Clostridiales\|Lachnospiraceae | 0.05 | 0.46 | 0.05 | 0.33 | 3.841 | 0.0463 |
| Clostridia\|Clostridiales\|Lachnospiraceae_otu203 | - | 0.29 | - | 0.28 | 3.888 | 0.0369 |

[1]Average abundances (expressed as percentages) as well as standard deviation are presented for each taxon. EXT = extensive grazing system; SEMI = semi-intensive grazing system

**Table S6.** Differentially abundant taxa in cheese core **(A)** in July and **(B)** in September between EXT (*n*=3) and SEMI (*n*=3) systems as determined by the LDA method[1].

**A**

| Taxa | Average abundance | | Standard deviation | | LDA score | pval |
|---|---|---|---|---|---|---|
| | EXT | SEMI | EXT | SEMI | | |
| Taxa more abundant in EXT | | | | | | |
| ***Firmicutes*** | | | | | | |
| Bacilli\|Lactobacillales\|Lactobacillaceae | 1.52 | 0.66 | 0.83 | 0.32 | 4.028 | 0.0495 |
| Bacilli\|Lactobacillales\|Lactobacillaceae\|Lactobacillus\| Lb. helveticus_otu27 | 0.62 | 0.09 | 0.42 | 0.07 | 3.436 | 0.0495 |
| Bacilli\|Lactobacillales\|Lactobacillaceae\|Lactobacillus\| Lb. parabuchneri_otu57 | 0.11 | 0.04 | 0.02 | 0.03 | 2.977 | 0.0495 |
| Bacilli\|Lactobacillales\|Lactobacillaceae\|Lactobacillus\| Lactobacillus spp._otu180 | 0.04 | 0.01 | 0.02 | 0.00 | 2.845 | 0.0495 |
| Bacilli\|Lactobacillales\|Lactobacillaceae\|Lactobacillus\| Lb. helveticus_otu229 | 0.06 | 0.01 | 0.05 | 0.01 | 2.524 | 0.0495 |
| Taxa more abundant in SEMI | | | | | | |
| ***Fusobacteria*** | | | | | | |
| Fusobacteriia\|Fusobacteriales\|Fusobacteriaceae\| Fusobacterium_ otu223 | 0.03 | 0.16 | 0.03 | 0.09 | 2.999 | 0.0495 |

**B**

| Taxa | Average abundance | | Standard deviation | | LDA score | pval |
|---|---|---|---|---|---|---|
| | EXT | SEMI | EXT | SEMI | | |
| Taxa more abundant in EXT | | | | | | |
| ***Proteobacteria*** | | | | | | |
| Gammaproteobacteria\|Enterobacteriales | 0.13 | - | 0.18 | - | 2.914 | 0.0369 |
| Gammaproteobacteria\|Enterobacteriales\|Enterobacteriaceae | 0.13 | - | 0.18 | - | 2.905 | 0.0369 |
| Gammaproteobacteria\|Pseudomonadales\| Pseudomonadaceae\|Pseudomonas\|Ps. putida_otu326 | 0.02 | - | 0.02 | - | 2.185 | 0.0369 |
| Taxa more abundant in SEMI | | | | | | |
| ***Firmicutes*** | | | | | | |
| Bacilli\|Lactobacillales\|Lactobacillaceae\|Lactobacillus\| Lb. curvatus_otu315 | - | 0.02 | - | 0.01 | 2.407 | 0.0339 |
| Bacilli\|Lactobacillales\|Streptococcaceae\|Streptococcus | 0.03 | 0.11 | 0.02 | 0.03 | 2.627 | 0.0495 |
| Bacilli\|Lactobacillales\|Streptococcaceae\|Lactococcus\| Lc. lactis_otu46 | 0.09 | 0.36 | 0.07 | 0.08 | 3.153 | 0.0495 |
| Bacilli\|Lactobacillales\|Streptococcaceae\|Lactococcus_otu139 | 0.02 | 0.07 | 0.01 | 0.03 | 2.440 | 0.0495 |
| Bacilli\|Lactobacillales\|Streptococcaceae\|Lactococcus_otu175 | 0.02 | 0.06 | 0.01 | 0.03 | 2.307 | 0.0495 |
| Bacilli\|Lactobacillales\|Streptococcaceae\|Lactococcus\| Lc. chungangensis/raffinolactis_otu48 | 0.07 | 0.30 | 0.06 | 0.09 | 3.048 | 0.0495 |
| Bacilli\|Lactobacillales\|Streptococcaceae\|Lactococcus_otu89 | 0.06 | 0.23 | 0.05 | 0.03 | 2.922 | 0.0495 |

[1]Average abundances (expressed as percentages) as well as standard deviation are presented for each taxon. EXT = extensive grazing system; SEMI = semi-intensive grazing system

**Table S7.** Differentially abundant taxa in cheese rind **(A)** in July and **(B)** in September between EXT ($n$=3) and SEMI ($n$=3) systems as determined by the LDA method[1].

**A**

| Taxa | Average abundance | | Standard deviation | | LDA score | pval |
|---|---|---|---|---|---|---|
| | EXT | SEMI | EXT | SEMI | | |
| Taxa more abundant in EXT | | | | | | |
| ***Firmicutes*** | | | | | | |
| Bacilli\|Lactobacillales\|Lactobacillaceae\|Lactobacillus\| Lb. helveticus_otu27 | 0.72 | 0.16 | 0.49 | 0.02 | 3.499 | 0.0495 |
| Bacilli\|Lactobacillales.\|Lactobacillaceae\|Lactobacillus\| Lb. helveticus_otu229 | 0.05 | 0.01 | 0.04 | 0.01 | 2.536 | 0.0495 |
| Taxa more abundant in SEMI | | | | | | |
| ***Bacteroidetes*** | | | | | | |
| Flavobacteriia\|Flavobacteriales\|Flavobacteriaceae\| Chryseobacterium spp._otu372 | - | 0.02 | - | 0.01 | 2.649 | 0.0495 |
| ***Firmicutes*** | | | | | | |
| Bacilli\|Lactobacillales\|Streptococcaceae\|Lactococcus\| Lc. lactis_otu214 | - | 0.01 | - | 0.01 | 2.770 | 0.0495 |
| Bacilli\|Lactobacillales\|Streptococcaceae\|Lactococcus\| Lc. chungangensis/raffinolactis_otu48 | 0.06 | 0.18 | 0.01 | 0.04 | 2.878 | 0.0495 |

**B**

| Taxa | Average abundance | | Standard deviation | | LDA score | pval |
|---|---|---|---|---|---|---|
| | EXT | SEMI | EXT | SEMI | | |
| Taxa more abundant in EXT | | | | | | |
| ***Proteobacteria*** | | | | | | |
| Gammaproteobacteria\|Enterobacteriales\| Enterobacteriaceae\|Escherichia\|E. coli_otu90 | 0.15 | 0.004 | 0.25 | 0.002 | 2.958 | 0.0495 |
| Taxa more abundant in SEMI | | | | | | |
| ***Actinobacteria*** | | | | | | |
| Actinobacteria\|Micrococcales\|Brevibacteriaceae\| Brevibacterium_otu94 | 0.26 | 0.41 | 0.06 | 0.06 | 2.969 | 0.0495 |
| ***Firmicutes*** | | | | | | |
| Bacilli\|Lactobacillales\|Streptococcaceae\|Lactococcus\| Lc. lactis_otu46 | 0.07 | 0.23 | 0.05 | 0.09 | 2.924 | 0.0495 |
| Bacilli\|Lactobacillales\|Streptococcaceae\|Lactococcus\| Lc. chungangensis/raffinolactis_otu48 | 0.06 | 0.31 | 0.05 | 0.14 | 3.135 | 0.0495 |
| Bacilli\|Lactobacillales\|Streptococcaceae\|Lactococcus_ otu89 | 0.04 | 0.21 | 0.03 | 0.08 | 3.006 | 0.0495 |

[1]Average abundances (expressed as percentages) as well as standard deviation are presented for each taxon. EXT = extensive grazing system; SEMI = semi-intensive grazing system

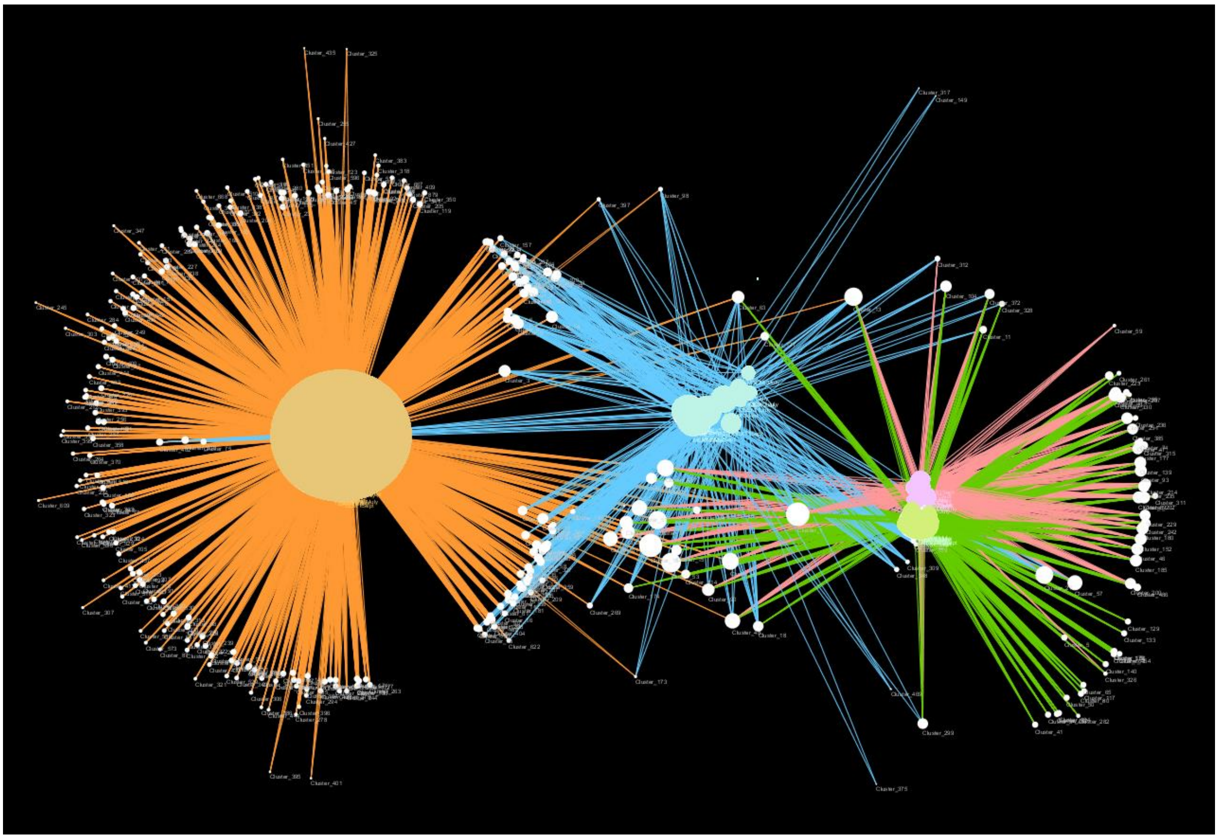

**Figure S3.** Network analysis from teat skin to ripened cheeses. The teat skin samples were coloured in orange, raw milk samples in blue, cheese core samples in pink, and cheese rind samples in green. The white nodes correspond to OTUs. The node size is proportional to the number of the edges (lines connecting the nodes). The OTUs detected in at least two samples of the same habitat were conserved.

**Table S8.** Average relative abundance (%) of 133 OTUs present in at least two habitats. The 9 OTUs detected in all habitats were framed.

Colour scale: 0.1% | 0.5% | 1% | 5% | 10% | 50%

| Taxa | N°otu | Teat skin (n=12) | Milk (n=12) | Core (n=12) | Rind (n=12) |
|---|---|---|---|---|---|
| ***Actinobacteria*** | | | | | |
| *Brevibacterium linens* | OTU4 | 0.01 | 0.06 | 0.02 | 12.8 |
| *Bifidobacterium pseudolongum* | OTU6 | 7.31 | 0.38 | | |
| *Corynebacterium marinum* | OTU8 | 5.87 | 1.58 | | |
| *Brevibacterium linens* | OTU11 | | 0.05 | | 1.87 |
| *Corynebacterium* spp. | OTU12 | 3.62 | 0.83 | | |
| *Brachybacterium paraconglomeratum/sacelli* | OTU15 | 0.01 | 0.04 | | 1.22 |
| *Corynebacterium* | OTU17 | 1.70 | 1.01 | | |
| *Bifidobacterium crudilactis/psychraerophilum* | OTU18 | | 10.7 | | 0.01 |
| *Dietzia maris* | OTU19 | 1.34 | 0.85 | | |
| *Corynebacterium callunae* | OTU20 | 1.37 | 0.86 | | |
| *Corynebacterium camporealensis* | OTU21 | 1.36 | 0.11 | | |
| *Corynebacterium* spp. | OTU22 | 1.28 | 0.24 | | |
| *Corynebacterium glutamicum* | OTU25 | 1.12 | 0.16 | | |
| *Corynebacteriumhumireducens* | OTU32 | 0.84 | 0.38 | | |
| *Rhodococcus* spp. | OTU33 | 0.04 | 6.10 | | |
| *Bifidobacterium choerinum* | OTU36 | 0.78 | 0.06 | | |
| *Brevibacterium* | OTU42 | 0.07 | | | 0.51 |
| *Brachybacterium sacelli* | OTU44 | 0.03 | | | 0.30 |
| *Bifidobacterium merycicum* | OTU52 | 0.44 | 0.3 | | |
| *Yaniella halotolerans* | OTU54 | 0.01 | | | 0.24 |
| *Nocardioides* | OTU55 | 0.51 | 0.03 | | |
| *Corynebacterium* | OTU56 | 0.61 | 0.03 | | |
| *Pseudoclavibacter helvolus* | OTU58 | 0.35 | 0.31 | | |
| *Arthrobacter* spp. | OTU66 | 0.36 | 0.14 | | |
| *Kocuria* spp. | OTU72 | 0.33 | 0.03 | | |
| *Atopobium* | OTU75 | 0.34 | 0.02 | | |
| *Corynebacterium lactis* | OTU76 | 0.63 | 0.02 | | |
| *Micrococcus luteus/terreus* | OTU78 | 0.39 | 0.12 | | |
| *Micrococcus* | OTU79 | 0.29 | 0.01 | | |
| *Tetrasphaera* | OTU91 | 0.29 | 0.02 | | |
| *Dietzia lutea* | OTU99 | 0.25 | 0.02 | | |
| *Rhodococcus* spp. | OTU100 | 0.02 | 2.25 | | |
| *Atopobium* | OTU112 | 0.38 | 0.02 | | |
| *Nocardioides solisilvae* | OTU120 | 0.27 | 0.02 | | |
| *Atopobium* | OTU124 | 0.27 | 0.02 | | |
| *Dietzia cercidiphylli* | OTU127 | 0.01 | 0.01 | | 0.09 |
| *Atopobium* | OTU130 | 0.27 | 0.01 | | |
| *Corynebacterium* | OTU137 | 0.17 | 0.02 | | |
| *Janibacter* spp. | OTU153 | 0.22 | 0.07 | | 0.02 |
| *Nocardioides daphniae* | OTU157 | 0.13 | 0.07 | | |
| *Kocuria rhizophila/salsicia* | OTU174 | 0.01 | 0.64 | | 0.01 |
| *Micrococcus* spp. | OTU234 | 0.09 | 0.04 | | |

| | | | | | |
|---|---|---|---|---|---|
| *Corynebacterium bovis* | OTU269 | 0.02 | 0.3 | | |
| *Corynebacterium variabile* | OTU328 | | 0.04 | | 0.02 |
| *Agreia* | OTU335 | 0.05 | 0.07 | | |
| *Rhodococcus* spp. | OTU397 | 0.01 | 0.21 | | |
| *Leucobacter* spp. | OTU404 | 0.10 | 0.16 | | |
| ***Bacteroidetes*** | | | | | |
| *Chryseobacterium* spp. | OTU372 | | 0.25 | 0.01 | 0.01 |
| ***Firmicutes*** | | | | | |
| *Lactococcus lactis* | OTU1 | 0.23 | 11.6 | 97.4 | 69.7 |
| *Romboutsia* | OTU3 | 14.6 | 1.85 | | |
| *Intestinibacter* | OTU7 | 6.42 | 0.87 | | |
| *Turicibacter* | OTU9 | 5.59 | 1.53 | | |
| *Peptoclostridium* | OTU10 | 5.58 | 0.30 | | |
| *Lactobacillus casei/paracasei* | OTU13 | 0.01 | 6.61 | 0.42 | 0.60 |
| *Staphylococcus haemolyticus/petrasii* | OTU16 | 2.37 | 0.80 | 0.10 | 0.25 |
| *Bacillus* spp. | OTU24 | 0.02 | 6.71 | | |
| *Aerococcus urinaeequi/viridans* | OTU26 | 1.21 | 0.04 | | |
| *Lactobacillus helveticus* | OTU27 | | 3.45 | 0.23 | 0.27 |
| *Bacillus* | OTU29 | 1.03 | 0.09 | | |
| *Jeotgalicoccus* spp. | OTU34 | 1.39 | 0.04 | | 0.08 |
| *Clostridium* spp. | OTU35 | 0.80 | 0.04 | | |
| *Acetitomaculum* | OTU37 | 0.73 | 0.05 | | |
| *Lactococcus lactis* | OTU39 | | | 0.42 | 0.21 |
| *Lactococcus lactis* | OTU46 | | | 0.16 | 0.11 |
| *Lactococcus chungangensis/raffinolactis* | OTU48 | 0.01 | 0.06 | 0.12 | 0.15 |
| *Macrococcus caseolyticus* | OTU51 | 0.29 | 0.25 | 0.06 | 0.10 |
| *Streptococcus porcinus/uberis* | OTU53 | 0.04 | 4.04 | 0.03 | 0.06 |
| *Lactobacillus parabuchneri* | OTU57 | | 0.16 | 0.06 | 0.27 |
| *Proteiniclasticum* | OTU60 | 0.52 | 0.07 | | |
| *Aerococcaceae* | OTU62 | 0.72 | 0.01 | | |
| *Enterococcus* spp. | OTU63 | 0.02 | 0.48 | | 0.11 |
| *Staphylococcus chromogenes* | OTU67 | 0.36 | 0.40 | 0.01 | |
| *Streptococcus dysgalactiae* | OTU81 | 0.12 | 0.26 | 0.02 | 0.03 |
| *Clostridium* | OTU85 | 0.27 | 0.04 | | |
| *Lachnospiraceae* | OTU88 | 0.24 | 0.03 | | |
| *Lactococcus* | OTU89 | | | 0.10 | 0.09 |
| *Lactococcus lactis* | OTU93 | | | 0.11 | 0.04 |
| *Turicibacter sanguinis* | OTU95 | 0.23 | 0.01 | | |
| *Facklamia* | OTU96 | 0.29 | 0.02 | | |
| *Staphylococcus equorum* | OTU114 | 0.02 | 0.01 | | 0.11 |
| *Ruminococcaceae* | OTU122 | 0.44 | 0.21 | | |
| *Marvinbryantia* | OTU132 | 0.30 | | | |
| *Mogibacterium* | OTU135 | 0.22 | 0.09 | | |
| *Lactococcus* | OTU139 | | | 0.04 | 0.03 |
| *Christensenellaceae* | OTU146 | 0.29 | 0.01 | | |
| *Lactococcus* | OTU152 | | | 0.07 | 0.05 |
| *Lachnospiraceae* | OTU156 | 0.10 | 0.04 | | |
| *Christensenellaceae* | OTU164 | 0.11 | 0.04 | | |
| *Lactococcus* | OTU175 | | | 0.04 | 0.04 |
| *Lactobacillus brevis* | OTU177 | | | 0.02 | 0.03 |

| | | | | | |
|---|---|---|---|---|---|
| *Lactobacillus* spp. | OTU180 | | | 0.02 | 0.03 |
| *Lachnospiraceae* | OTU181 | 0.15 | 0.01 | | |
| *Lactococcus* | OTU185 | | | 0.07 | 0.06 |
| *Ruminococcaceae* | OTU186 | 0.18 | 0.01 | | |
| *Ruminococcaceae* | OTU197 | 0.08 | 0.13 | | |
| *Lactococcus lactis* | OTU200 | | | 0.03 | 0.02 |
| *Lactobacillus* spp. | OTU202 | | | 0.03 | 0.03 |
| *Lachnospiraceae* | OTU203 | 0.20 | 0.07 | | |
| *Ruminococcaceae* | OTU206 | 0.15 | 0.02 | | |
| *Streptococcus pluranimalium* | OTU209 | 0.06 | 0.32 | | |
| *Lactococcus lactis* | OTU214 | | | 0.02 | 0.01 |
| *Lactobacillus helveticus* | OTU229 | | | 0.02 | 0.02 |
| *Lactobacillus* spp. | OTU235 | | | 0.02 | 0.02 |
| *Lactococcus* | OTU242 | | | 0.01 | 0.01 |
| *Lactobacillus parabuchneri* | OTU251 | | | 0.01 | 0.04 |
| *Christensenellaceae* | OTU258 | 0.12 | 0.10 | | |
| *Planomicrobium* | OTU295 | | | 0.02 | 0.01 |
| *Macrococcus caseolyticus* | OTU311 | | | 0.02 | 0.02 |
| *Lactobacillus curvatus* | OTU315 | | | 0.01 | 0.01 |
| *Lachnospiraceae* | OTU327 | 0.06 | 0.02 | | |
| *Streptococcus* | OTU330 | | | 0.02 | 0.01 |
| *Ruminococcaceae* | OTU339 | 0.06 | 0.06 | | |
| *Paenibacillus* spp. | OTU359 | 0.04 | 0.03 | | |
| *Lactococcus lactis* | OTU385 | | | 0.02 | 0.01 |
| *Christensenellaceae* | OTU402 | 0.07 | | | |
| *Fastidiosipila* | OTU417 | 0.03 | | 0.01 | 0.01 |
| ***Fusobacteria*** | | | | | |
| *Fusobacterium* | OTU223 | | | 0.05 | 0.01 |
| ***Proteobacteria*** | | | | | |
| *Sphingomonas* spp. | OTU14 | 0.24 | 19.6 | | |
| *Paracocccus chinensis* | OTU61 | 0.67 | 0.16 | | |
| *Escherichia coli* | OTU90 | 0.01 | 0.01 | 0.05 | 0.07 |
| *Cupriavidus gilardii* | OTU98 | 0.01 | 1.43 | | |
| *Sphingomonas* spp. | OTU101 | 0.20 | 0.41 | | |
| *Brevundimonas* spp. | OTU103 | 0.02 | 1.11 | | |
| *Pseudomonas* spp. | OTU104 | | 0.73 | 0.01 | 0.04 |
| *Paracocccus* spp. | OTU107 | 0.36 | 0.01 | | |
| *Sphingomonadaceae changbaiensis* | OTU173 | 0.01 | 0.58 | | |
| *Bradyrhizobium* spp. | OTU267 | 0.04 | 0.11 | | |
| *Rhizobium* | OTU288 | 0.08 | 0.04 | | |
| *Acinetobacter guillouiae* | OTU299 | | 0.06 | 0.01 | 0.01 |
| *Acinetobacter* | OTU312 | | 0.31 | 0.02 | |
| *Pseudomonas putida* | OTU326 | | | 0.01 | 0.02 |
| *Enterobacteriaceae* | OTU486 | | | 0.01 | 0.02 |
| *Bosea* | OTU622 | 0.02 | 0.04 | | |

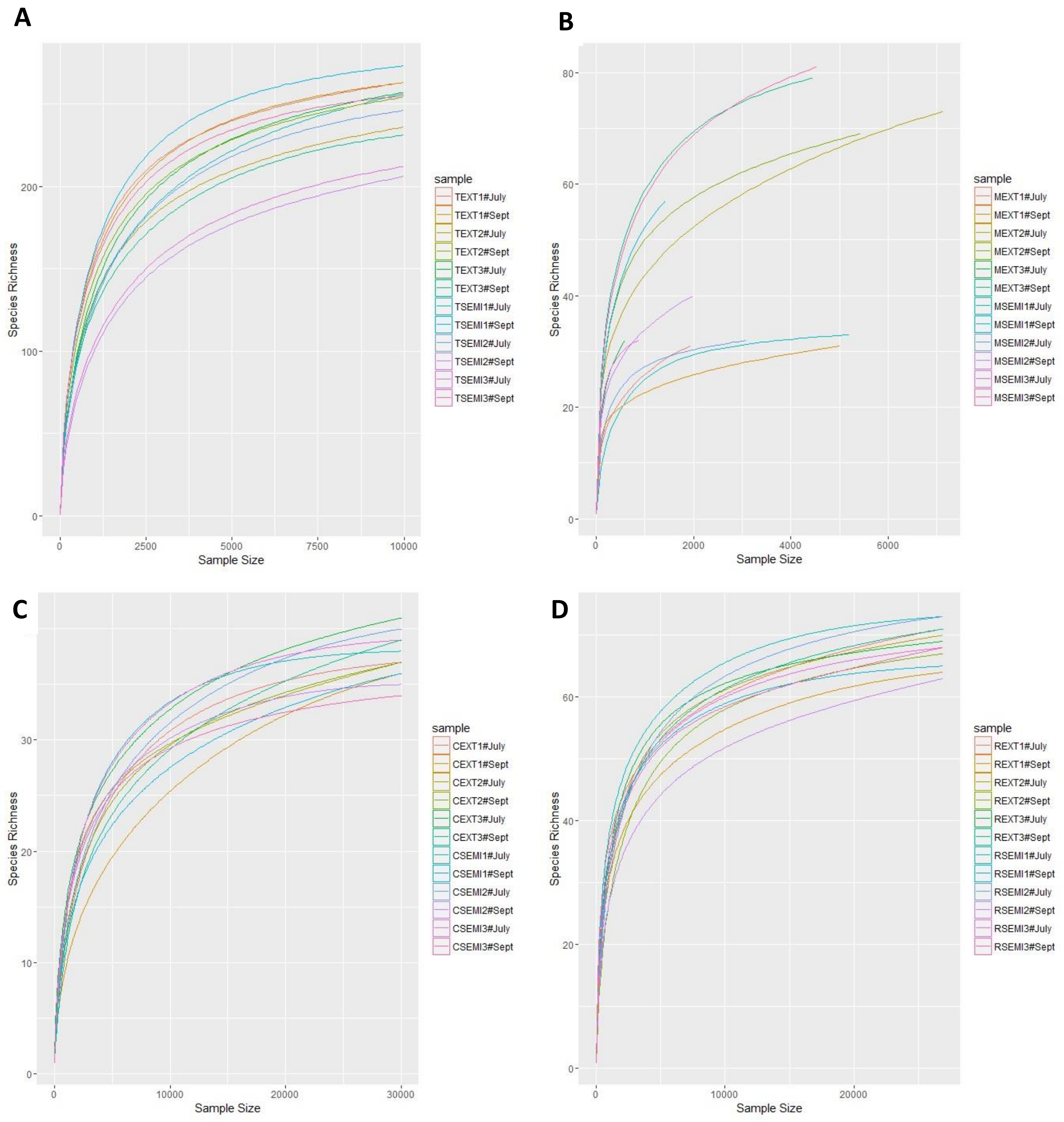


**Figure S4.** Rarefaction curves from the V3-V4 region of the 16S rRNA from teat skin (A), raw milk (B), cheese core (C), and cheese rind (D) samples.
